# Lateral hypothalamic neurotensin neurons promote arousal and hyperthermia

**DOI:** 10.1371/journal.pbio.3000172

**Published:** 2019-03-20

**Authors:** Fumito Naganuma, Daniel Kroeger, Sathyajit S. Bandaru, Gianna Absi, Joseph C. Madara, Ramalingam Vetrivelan

**Affiliations:** 1 Department of Neurology, Beth Israel Deaconess Medical Center, Boston, Massachusetts, United States of America; 2 Division of Sleep Medicine, Harvard Medical School, Boston, Massachusetts, United States of America; 3 Division of Pharmacology, Faculty of Medicine, Tohoku Medical and Pharmaceutical University, Sendai, Japan; 4 Division of Endocrinology, Diabetes and Metabolism, Department of Medicine, Beth Israel Deaconess Medical Center, Boston, Massachusetts, United States of America; Williams College, UNITED STATES

## Abstract

Sleep and wakefulness are greatly influenced by various physiological and psychological factors, but the neuronal elements responsible for organizing sleep-wake behavior in response to these factors are largely unknown. In this study, we report that a subset of neurons in the lateral hypothalamic area (LH) expressing the neuropeptide neurotensin (Nts) is critical for orchestrating sleep-wake responses to acute psychological and physiological challenges or stressors. We show that selective activation of Nts^LH^ neurons with chemogenetic or optogenetic methods elicits rapid transitions from non-rapid eye movement (NREM) sleep to wakefulness and produces sustained arousal, higher locomotor activity (LMA), and hyperthermia, which are commonly observed after acute stress exposure. On the other hand, selective chemogenetic inhibition of Nts^LH^ neurons attenuates the arousal, LMA, and body temperature (Tb) responses to a psychological stress (a novel environment) and augments the responses to a physiological stress (fasting).

## Abstract 2

A neurotensin-producing subset of neurons in the lateral hypothalamus promote arousal and thermogenesis; these neurons are necessary for appropriate sleep-wake and body temperature responses to various stressors.

## Author summary

Adjusting sleep-wake behavior in response to environmental and physiological challenges may not only be of protective value, but can also be vital for the survival of the organism. For example, while it is crucial to increase wake to explore a novel environment to search for potential threats and food sources, it is also necessary to decrease wake and reduce energy expenditure during prolonged absence of food. In this study, we report that a subset of neurons in the lateral hypothalamic area (LH) expressing the neuropeptide neurotensin (Nts) is critical for orchestrating sleep-wake responses to such challenges. We show that brief activation of Nts^LH^ neurons in mice evokes immediate arousals from sleep, while their sustained activation increases wake, locomotor activity, and body temperature for several hours. In contrast, when Nts^LH^ neurons are inhibited, mice are neither able to sustain wake in a novel environment nor able to reduce wake during food deprivation. These data suggest that Nts^LH^ neurons may be necessary for generating appropriate sleep-wake responses to a wide variety of environmental and physiological challenges.

The lateral hypothalamic area (LH) is considered to be a vital center for regulating sleep-wake behavior [1–3], but the specific neural elements involved in this process still remain elusive. Like other brain regions, the LH is heterogeneous and contains numerous neurochemically distinct populations of neurons [1]. While the majority of LH neurons use glutamate and GABA as neurotransmitters, they also express several neuropeptides, including orexin and melanin-concentrating hormone (MCH). Nonspecific electrolytic or neurotoxic lesions of the LH decrease the amounts of daily wakefulness and the ability to maintain wake [4]. Similarly, pharmacological inhibition of the LH increases non-rapid eye movement (NREM) sleep [5]. Although these findings indicate that one of the functions of the LH as a whole is to promote wakefulness, specific subsets of neurons in this area may play distinct roles in sleep-wake regulation. In vivo electrophysiological studies identified neurons that are maximally active during specific states of sleep-wake [6–9]. Similarly, selective activation of different subsets of LH neurons produces distinct sleep-wake changes. For example, activation of orexin and GABAergic neurons increase wakefulness [10–13], while activation of MCH neurons increase rapid eye movement (REM) sleep [14, 15]. Interestingly, a subset of GABAergic neurons within the LH also has been shown to increase NREM sleep in mice [16]. Thus, various subsets of LH neurons may play specific and independent roles in sleep-wake control.

In addition to orexin and MCH neurons, the LH comprises a large population of peptidergic neurons neurochemically defined by the presence of neurotensin (Nts) [17–20], whose role in sleep-wake has not been clearly defined. The available data thus far suggest that these neurons may be involved in wake promotion. For example, Nts^LH^ neurons heavily project to dopaminergic neurons in the ventral tegmental area (VTA) [21, 22], and microinjections of Nts into the VTA increase motor activity in mice [23–25]. Nts^LH^ neurons also project locally to orexin neurons [22], and application of Nts in the LH increases the firing of LH orexin neurons in brain slices [26]. Importantly, systemic application of an Nts antagonist in wild-type (WT) mice increases NREM sleep, although this antagonism is not specific to Nts^LH^ neurons [26]. Because both LH orexin and VTA dopaminergic neurons play important roles in wake promotion [12, 27] and are activated by Nts [26, 28–30], we hypothesized that Nts^LH^ neurons may form another excitatory wake-promoting cell group within the LH.

To test this hypothesis and to assess the functional role of Nts^LH^ in sleep-wake, locomotor activity (LMA), and body temperature (Tb) regulation, we first specifically activated the Nts^LH^ neurons and studied changes in sleep-wake, LMA, and Tb. We found that the Nts^LH^ neurons produced a hyperarousal state, comprising uninterrupted wake, hyperactivity, and hyperthermia, commonly observed in response to acute stress [31]. Therefore, we then inhibited Nts^LH^ neurons and studied sleep-wake, LMA, and Tb responses to psychological and physiological challenges (stress): a novel environment and fasting, respectively.

## Results

### Experiment 1: Characterization of Nts neurons in the LH as distinct neuronal population

We first analyzed the distribution of Nts neurons in the LH by generating transgenic mice expressing green fluorescent protein (GFP) exclusively in Nts neurons (Nts-Cre::L10-GFP; henceforth “Nts-GFP” mice; see Methods section). We found that Nts neurons are densely packed in the perifornical LH, intermingled with orexin and MCH neurons. We also observed another dense population of Nts neurons in the subthalamic nucleus of the basal ganglia located dorsolateral to the LH and a less-dense population in the dorsomedial hypothalamus (DMH) lying medial to the LH. However, this study specifically focuses on the Nts neurons in the LH (Nts^LH^), and all brain injections were aimed at this population.

**Fig 1:**
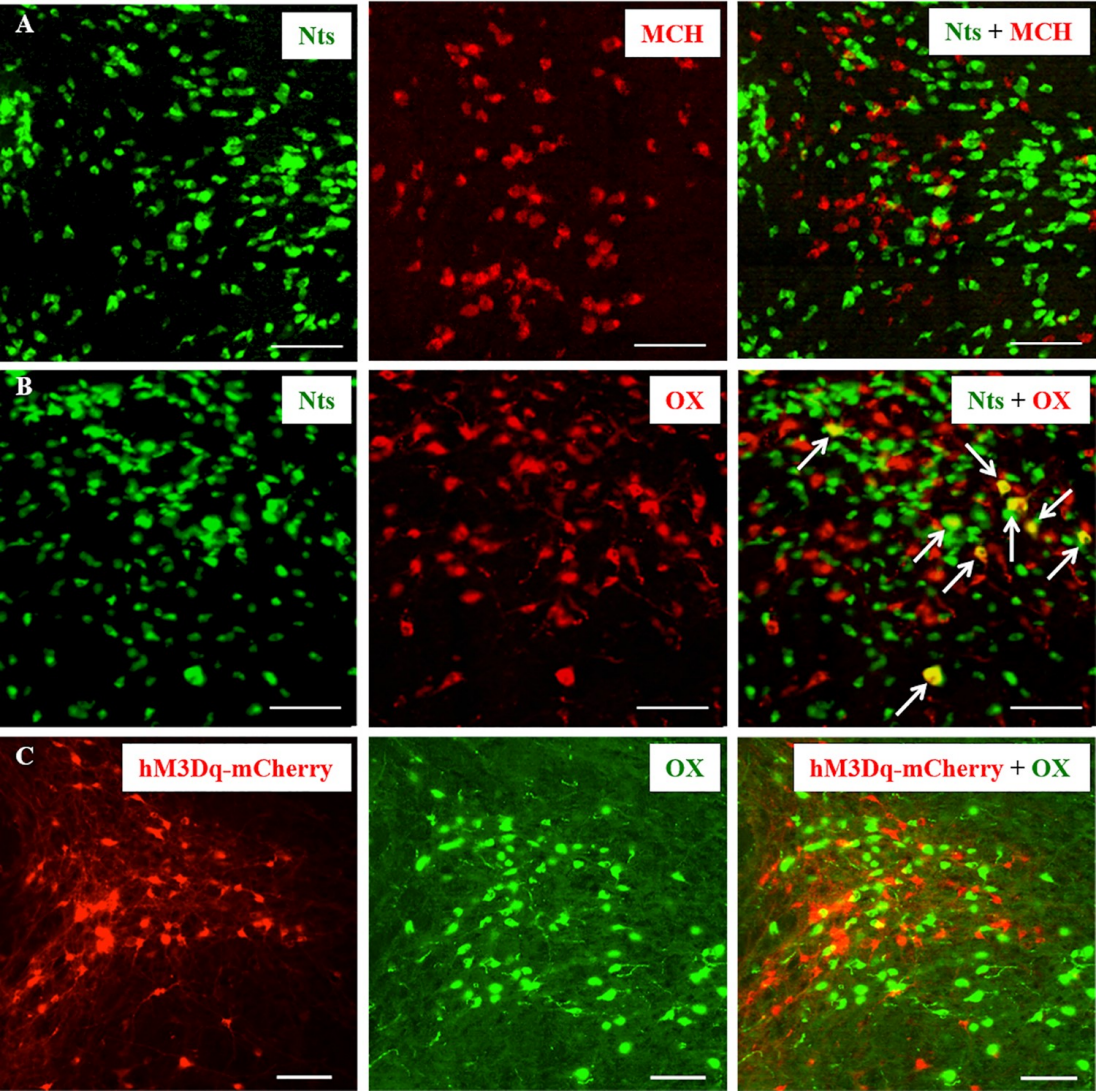
Nts neurons in the LH are distinct from orexin and MCH neurons. (**A, B**) Representative brain sections from Nts-GFP mice labeled for MCH (red cells in A) orexin (red cells in B). Nts neurons (green) on these sections were visualized by their native expression of GFP. About 4% of Nts neurons were also labeled for orexin (arrows), while none were labeled for MCH. (**C**) Representative brain section from one Nts-Cre mouse injected with AAV-hM3Dq into the LH, immunohistochemically labeled for mCherry (red; to label Cre-expressing Nts neurons) and orexin (green). None of the mCherry+ neurons were labeled for orexin, indicating that Nts and orexin are not colocalized in the same neurons. Scale bar = 100 μm. GFP, green florescent protein; LH, lateral hypothalamus; MCH, melanin-concentrating hormone; Nts, neurotensin; OX, orexin.

As Nts neurons were found densely packed in the perifornical LH region, we examined whether these neurons co-express MCH or orexin by immunolabelling brain sections from Nts-GFP mice (*n* = 6) for MCH and orexin. We found that none of the GFP+ neurons were labeled for MCH, whereas 3.7 ± 0.8% of GFP+ neurons were labeled for orexin (Fig 1A and 1B), indicating that a small fraction of Nts neurons may express orexin. To determine whether this overlap between Nts and orexin expression in LH neurons is an artifact of developmental expression of Nts/Cre (the Nts-GFP mouse might express GFP congenitally), we stereotaxically injected a Cre-dependent adeno-associated viral (AAV) vector containing mCherry (AAV8-hSyn-DIO-hM3Dq-mCherry, henceforth “AAV-hM3Dq”; University of North Carolina Vector core, United States; see below) into the LH (anteroposterior: −1.7 mm, ventral: 5.1 mm, lateral: ±1.1 mm) of adult (8 wk old) Nts-Cre mice (*n* = 4). Six weeks after the injections, we perfused the mice and immunolabeled the brain sections for mCherry (to label virally transfected, Cre-expressing Nts neurons) and orexin (Fig 1C). In these brain sections, we did not find any double-labeled neurons in the LH, indicating that Nts^LH^ neurons are a distinct population from orexin neurons, but some of them may produce orexin during development.

### Experiment 2: Anterograde projections of Nts^LH^ neurons

**Fig 2:**
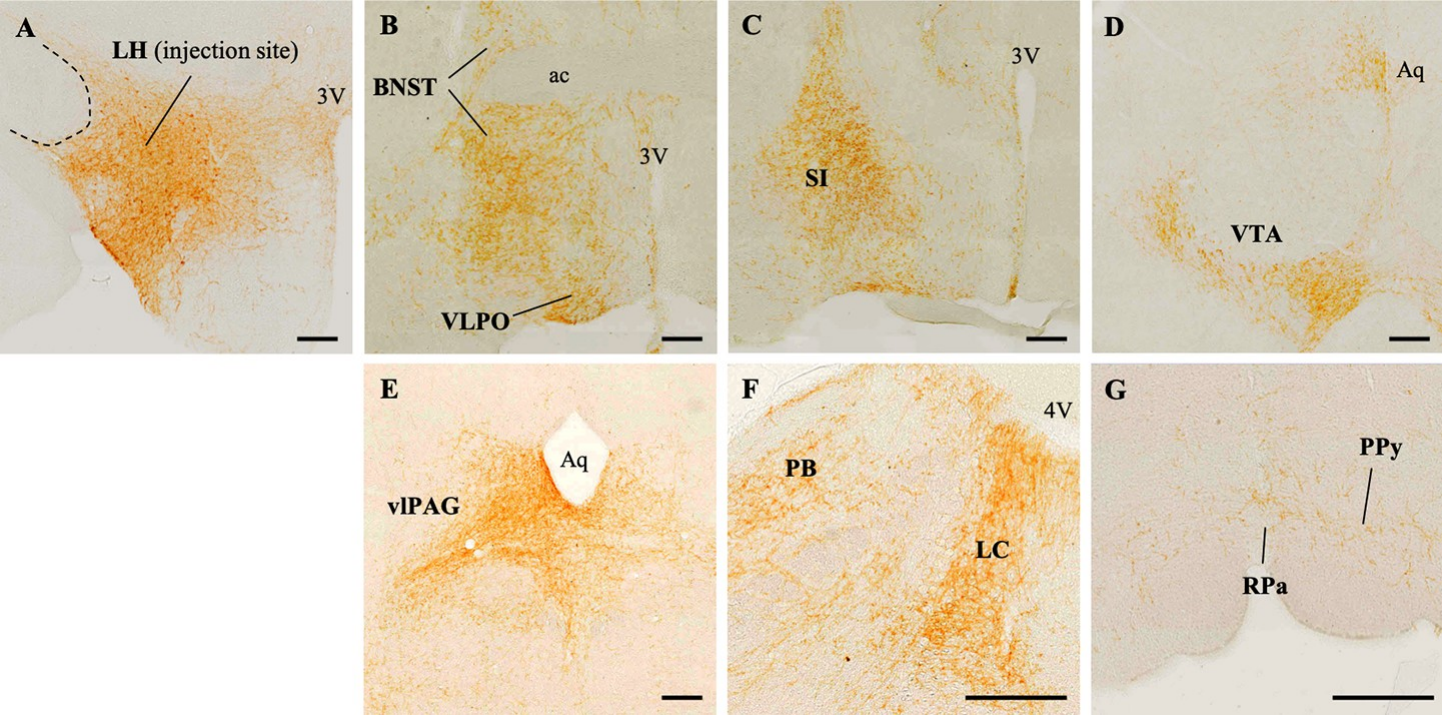
Anterograde projections of Nts^LH^ neurons. **(A)** AAV-ChR2 injection site represented by mCherry-labeled (brown) neurons in the LH. (**B-G**) mCherry-labeled terminals in the target regions. Scale bar = 200 μm. ac, anterior commissure; Aq, cerebral aqueduct; BNST, bed nucleus of stria terminalis; LC, locus coeruleus; LH, lateral hypothalamus; Nts, neurotensin; OC, optic chiasm; PB, parabrachial nucleus; PPy, parapyramidal region; RPa, raphe pallidus; SI, substantia innominata; vlPAG, ventrolateral periaqueductal gray; VLPO, ventrolateral preoptic area; VTA, ventral tegmental area; 3V, third ventricle; 4V, fourth ventricle.

We next examined the projections of Nts^LH^ neurons using conditional anterograde tracing to understand the neuronal targets through which Nts^LH^ neurons may regulate sleep-wake and Tb. We unilaterally microinjected a Cre-dependent AAV coding for channelrhodospsin-2 (ChR2) and the fluorescent tag mCherry (AAV8-EF1α-DIO-ChR2-mCherry, henceforth “AAV-ChR2”; University of North Carolina Vector core, US) [32, 33] into the LH of Nts-Cre mice (*n* = 6). Six weeks after the injections, we perfused the mice and processed the brain sections for immunohistochemical labeling of mCherry to identify ChR2-expressing Nts neurons and their axon terminals. AAV-ChR2 injections in three out of six mice were restricted to the LH (Fig 2A) without spread to adjacent regions, including the subthalamic nucleus or DMH, and only these cases were used to identify Nts^LH^ projections. A high density of Nts terminals were found in the VTA, ventrolateral periaqueductal gray (vlPAG), parabrachial nucleus (PB), locus coeruleus (LC), retrorubral region, substantia innominata, diagonal band of Broca, ventral pallidum, nucleus accumbens, raphe pallidus (RPa), parapyramidal region (Ppy), and lateral preoptic area (Fig 2B–2G). We observed a moderate density of Nts terminals in the dorsolateral septum and supramamillary nucleus. While all Nts^LH^ projections were predominantly ipsilateral, we also observed some less-dense contralateral projections in many of the target regions.

### Experiment 3: Optogenetic activation of Nts^LH^ neurons causes NREM-to-wake transitions

**Fig 3:**
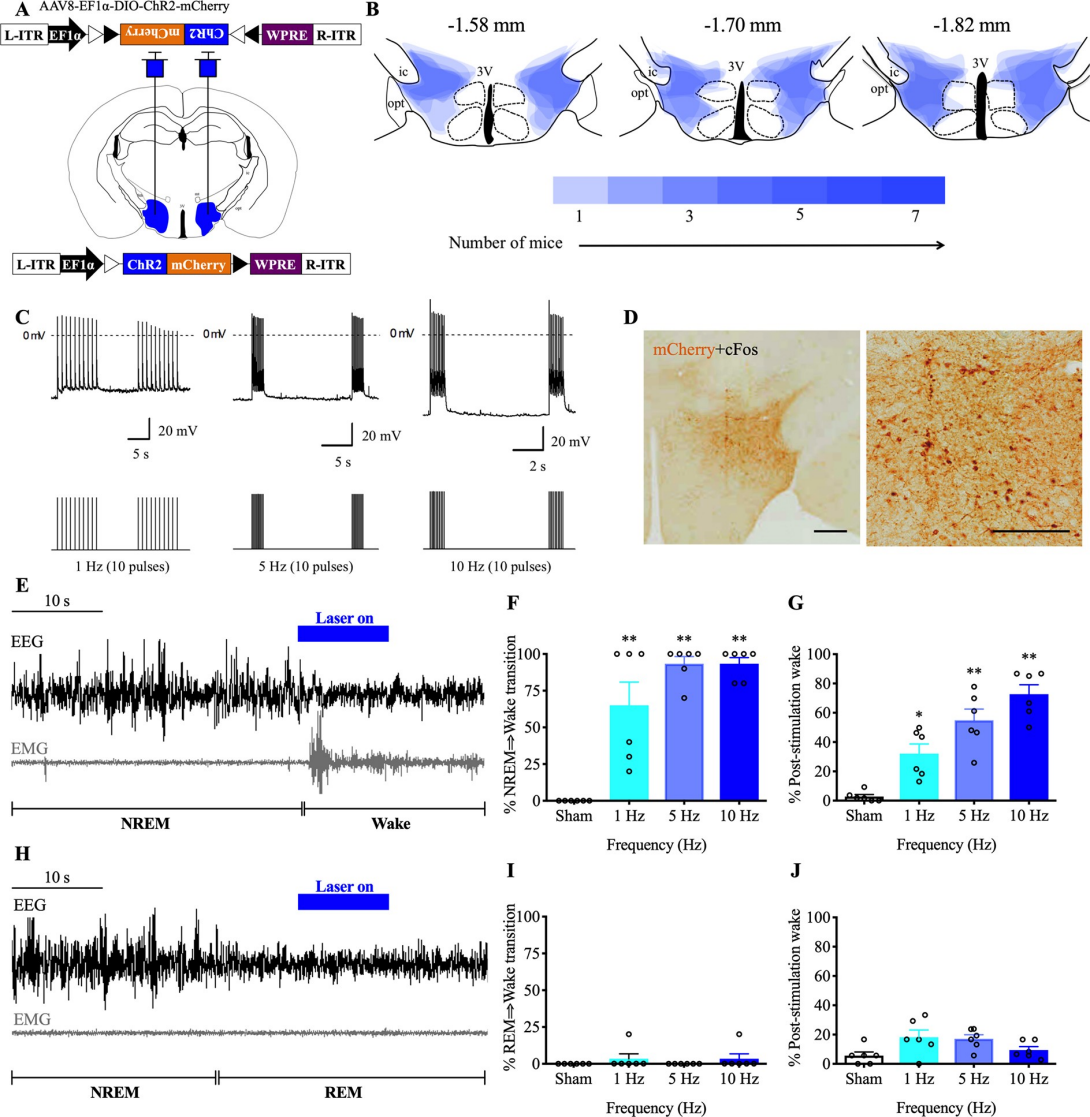
Brief optogenetic activation of Nts^LH^ neurons causes rapid arousal from NREM sleep. (**A**) Schematic representation of AAV-ChR2 injections and implantation of optical fibers. (**B**) Heat map of viral injection sites in the LH at three levels (anteroposterior: −1.58, −1.70, and −1.82 mm from bregma [36]); darker shades of blue represent more overlap of injection sites in different mice. (**C**) Ex vivo photostimulation (473-nm laser light; 10-ms pulses at 1, 5, and 10 Hz) reliably evoked frequency-dependent action potentials in mCherry-expressing Nts^LH^ neurons (*n* = 4) in brain slices; dotted line indicates the 0-mV level; laser stimulations are represented in the lower panel. (**D**) Blue laser stimulations (5 Hz, 10 ms) for 2 h in Nts-Cre mice injected with AAV-ChR2 resulted in robust cFos expression (black nuclei) in ChR2-mCherry–expressing neurons (brown) in the LH. Scale bar = 200 μm. Representative EEG/EMG traces demonstrating rapid transitions to wake upon photostimulations (5 Hz for 10 s) of Nts^LH^ neurons during NREM sleep (**E**) but not during REM sleep (**H**). Laser stimulations are marked in blue above the traces. Percentage of successful transitions to wake upon photostimulations (1, 5 and 10 Hz, 10 trials each) during NREM (**F**) or REM sleep (**I**). Percentage of wake during the 30-s period immediately after the cessation of photostimulations during NREM (**G**) or REM sleep (**J**). All data are mean ± SEM. *P < 0.05, **P < 0.01; one-way ANOVA followed by Tukey multiple comparisons test (*n* = 7 mice: *F* = 25.16, *P* < 0.0001). The underlying data for this figure are available from the Open Science Framework (https://osf.io/nmrpq/). ChR2, channelrhodospsin-2; EEG, electroencephalography; EMG, electromyography; L-ITR, left inverted terminal repeat; LH, lateral hypothalamic area; NREM, non-rapid eye movement; Nts, neurotensin; R-ITR, right inverted terminal repeat; REM, rapid eye movement.

We used optogenetic tools to activate Nts^LH^ neurons and investigated their role in sleep-wake control. We stereotaxically injected AAV-ChR2 bilaterally into the LH (anteroposterior: −1.7 mm, ventral: 5.1 mm, lateral: ±1.1 mm; Fig 3A) of Nts-Cre mice (*n* = 7) and implanted them with bilateral optical fibers (targeting 0.2 mm dorsal to the LH) for illumination with blue laser light [33], electrodes for recording electroencephalography (EEG) and electromyography (EMG) [34] and telemetry transmitters [35] for recording Tb and LMA.

Injections of AAV-ChR2 into the LH of Nts-Cre mice resulted in robust expression of ChR2-mCherry in Nts^LH^ neurons. In contrast, injections of the same AAV into the LH of WT littermates did not result in any expression of mCherry, indicating the Cre dependency of the AAV-ChR2. The AAV injections in Nts-Cre mice were largely restricted to the LH and zona incerta, with little or no spread medially into the DMH (Fig 3B).

We first tested the response of Nts^LH^ neurons to photo-illumination using ex vivo whole cell current clamp recordings. Illumination with blue laser light (473 nm at 1, 5, and 10 Hz) evoked action potentials in ChR2-mCherry–expressing Nts^LH^ neurons in a frequency-dependent manner (Fig 3C). In vivo, 5-Hz stimulation for 2 h prior to perfusion caused robust cFos expression in mCherry-expressing Nts^LH^ neurons, demonstrating that blue light illumination consistently drives activity in these neurons (Fig 3D).

To assess whether optogenetic activation of Nts^LH^ neurons influences sleep-wake states, we applied blue laser light pulses (473 nm, 10 ms) with different frequencies for 10 s specifically during NREM or REM sleep. We applied laser stimulations at frequencies of 1, 5, and 10 Hz (10 mW light power at the tip of the optical fibers) after either 30 s of stable NREM sleep or 10 s of stable REM sleep during the light period (from 10:00 AM to 5:00 PM). We applied 10 photostimulations of each frequency for each state. Photostimulations during NREM sleep in AAV-ChR2–injected Nts-Cre mice resulted in rapid transition to wake (Fig 3E). On average, about 65% of 1-Hz stimulations and 93% of 5-Hz and 10-Hz stimulations during NREM sleep produced a rapid (within 1–5 s of light pulse onset) arousal response (Fig 3F), characterized by EEG desynchronization, EMG activation, and behavioral wakefulness in Nts-Cre mice (Fig 3E). In addition, the amount of wakefulness during the 30-s period immediately after cessation of photostimulations showed a frequency-dependent increase, indicating that higher stimulation frequencies produce more rapid and longer-lasting wake responses (Fig 3G). In contrast, photostimulation during REM sleep had no effect on sleep-wake or EEG/EMG activity in Nts-Cre mice (Fig 3H–3J). Mice remained undisturbed and REM sleep continued during the entire stimulation period (Fig 3H and 3I). These findings demonstrate that activation of Nts^LH^ neurons rapidly trigger NREM-wake but not REM-wake transitions.

### Experiment 4: Photostimulation of Nts^LH^ neurons cause hyperthermia

**Fig 4:**
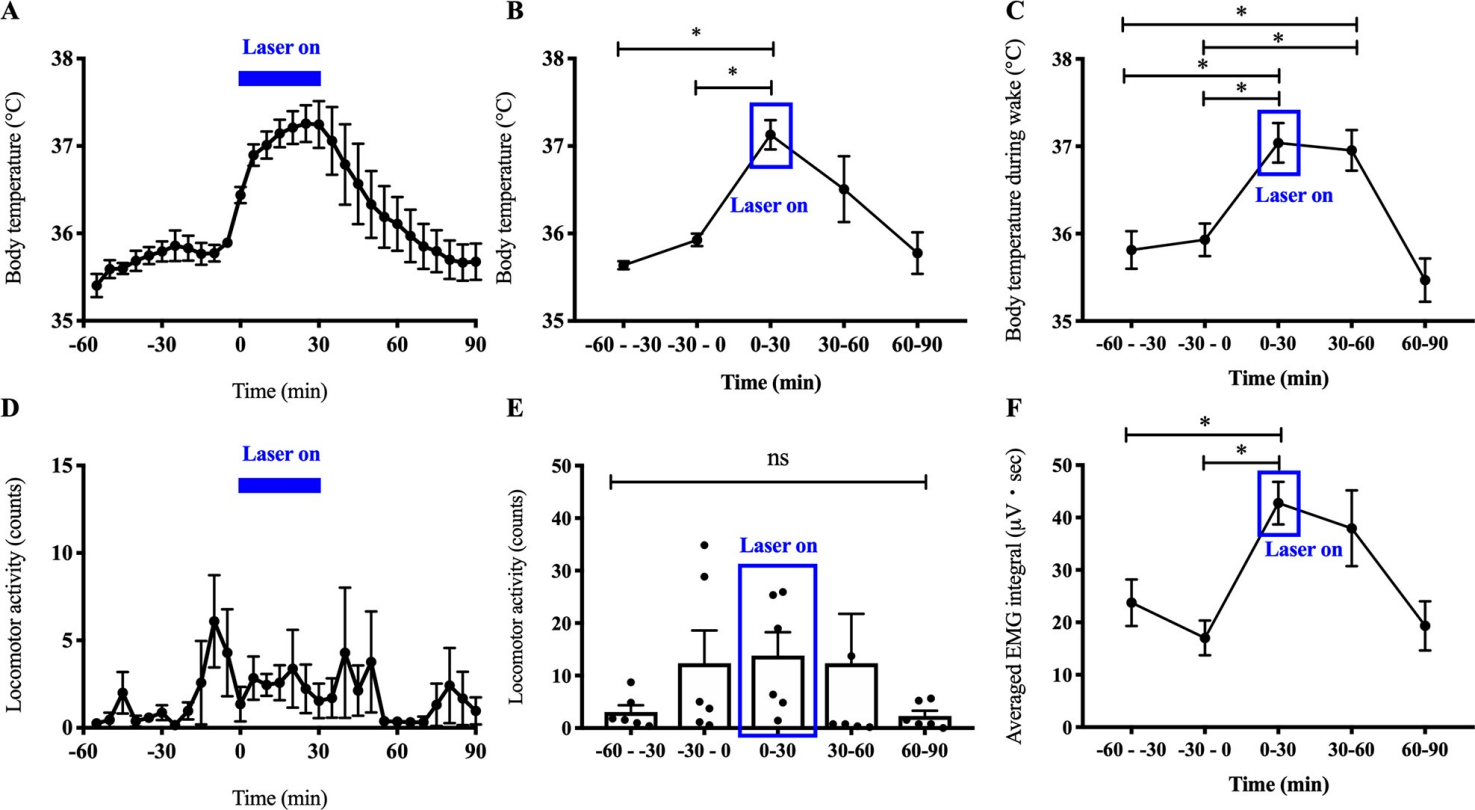
Subchronic optogenetic activation of Nts^LH^ neurons causes hyperthermia. Mean Tb and LMA counts in 5-min (**A, D**) and 30-min (**B, E**) intervals during the 1 h immediately before, 30 min during, and 1 h after photostimulation (5-Hz, 10-ms pulses) of Nts^LH^ neurons. Mean Tb specifically during wakefulness (**C**) and EMG integral (**F**) in 30-min bins during the same period. All data are mean ± SEM. *P < 0.05; one-way ANOVA followed by Tukey multiple comparisons test (n = 6–7 mice: for Tb, *F* = 8.18, *P* = 0.0004; for Tb_wake_, *F* = 10.21, *P* = 0.0001; for LMA, *F* = 1.04, *P* = 0.41; for EMG integral, *F* = 5.45, *P* = 0.0039). The underlying data for this figure are available from the Open Science Framework (https://osf.io/nmrpq/). EMG, electromyography; LH, lateral hypothalamic area; LMA, locomotor activity; Nts, neurotensin; Tb, body temperature.

Although brief 10-s activations of Nts^LH^ neurons are sufficient to evoke NREM-wake transitions, they are not sufficient to induce detectable changes in Tb or LMA. We therefore applied a continuous 5-Hz stimulation (10-ms pulse) for 30 min during the light period (at 10:00 AM) in Nts-Cre mice injected with AAV-ChR2. We observed a significant increase in Tb (1.35 ± 0.17°C increase) during the 30-min stimulation period (Fig 4A and 4B) when compared with 30 min prior. Tb began to drop almost immediately upon cessation of the stimulation and returned to baseline within the next approximately 30 min (Fig 4A). Because the photostimulations evoked immediate arousal in all mice (except in one mouse that was in REM sleep when the photostimulations began), and the mice were awake during the entire stimulation period (98.78% ± 1.22% versus 18.89% ± 6.44% during the 30 min prior; *P* < 0.001), we calculated the average Tb specifically during wake episodes (Tb_wake_). We found that the Tb_wake_ during the photostimulations was significantly higher (1.11 ± 0.3°C increase; *P* = 0.016) than Tb_wake_ during the 30 min prior to stimulations (Fig 4C), suggesting that the hyperthermia was not a mere coincidence of wake transitions. On the other hand, total LMA counts did not differ across these periods (Fig 4D and 4E), although the EMG activity (measured as integral EMG) during the photostimulations was 151% higher than prestimulation values (*P* = 0.004; one-way ANOVA; Fig 4F). The temporal correlation between the photostimulations and Tb on the one hand and the increase in Tb and EMG activity, even without substantial increase in LMA, on the other hand suggests that Nts^LH^ neurons may participate in thermogenesis.

### Experiment 5: Chemoactivation of Nts^LH^ neurons induces sustained wake and hyperthermia

**Fig 5:**
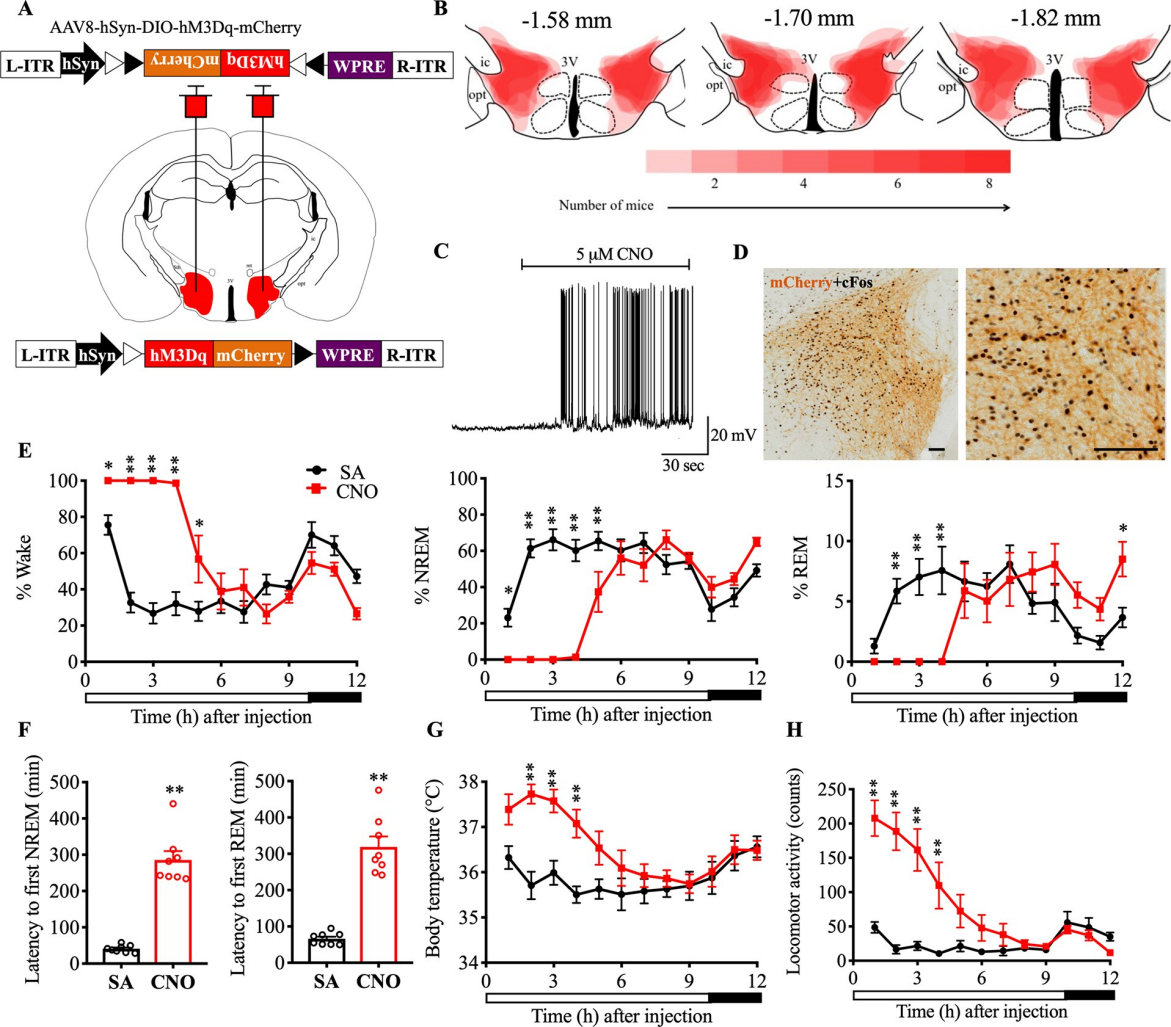
Chemoactivation of Nts^LH^ neurons causes sustained arousal and hyperthermia. (**A**) Schematic representation of AAV injections. (**B**) Heat map of AAV-hM3Dq injection sites in the LH at three levels (anteroposterior: −1.58, −1.7, and −1.82 mm [36]); darker shades of red indicate more overlap between injection sites of different mice. (**C**) Ex vivo bath application of CNO (5 μM) induced depolarization and increased action potentials in mCherry-expressing Nts^LH^ neurons (*n* = 5) in brain slices; (**D**) CNO (0.3 mg/kg; i.p.) induced robust cFos expression (black nuclei) in hM3Dq-mCherry–expressing neurons in the LH (brown). Hourly percentages of different sleep-wake states (**E**), mean Tb (**G**), and total LMA counts (**H**) for 12 h after saline or CNO (0.3 mg/kg) administration at 9:50 AM in Nts-Cre mice injected with AAV-hM3Dq into the LH. Two-way RM ANOVA for “time” and “compound injected,” followed by Sidak post hoc test (*n* = 7–8 mice; for wake: interaction *F*(11,84) = 18.47, *P* < 0.0001, compound injected *F*(11, 84) = 15.79, *P <* 0.0001, time *F*(1, 84) = 54.81, *P* < 0.0001; for NREM: interaction *F*(11, 84) = 17.69, *P* < 0.0001, compound injected *F*(11, 84) = 14.53, *P <* 0.0001, time *F*(1, 84) = 62.28, *P* < 0.0001; for REM: interaction *F*(11,84) = 8.64, *P* < 0.0001, compound injected *F*(11, 84) = 3.307, *P* = 0.0008, time *F*(1, 84) = 2.71, *P* = 0.10; for Tb: interaction *F*(11,60) = 5.33, *P* < 0.0001, compound injected *F*(11, 60) = 3.26, *P* = 0.0015, time *F*(1, 60) = 64.98, *P* < 0.0001; for LMA: interaction *F*(11,84) = 11.51, *P* < 0.0001, compound injected *F*(11, 84) = 10.05, *P <* 0.0001, time *F*(1, 84) = 80.07, *P* < 0.0001). Latency to NREM and REM sleep (**F**) after saline and CNO injections. Mann-Whitney test (n = 8 mice: for NREM: *P* = 0.0002; for REM: *P* = 0.0002). Data are mean ± SEM. *P < 0.05, **P < 0.01. The underlying data for this figure are available from the Open Science Framework (https://osf.io/nmrpq/). AAV, adeno-associated virus; CNO, clozapine-n-oxide; hSyn, human Synapsin-1; i.p., intraperitoneal; L-ITR, left inverted terminal repeat; LH, lateral hypothalamic area; LMA, locomotor activity; NREM, non-rapid eye movement; Nts, neurotensin; R-ITR, right inverted terminal repeat; REM, rapid eye movement; RM, repeated measures; SA, saline; Tb, body temperature; WPRE, woodchuck hepatitis virus posttranscriptional control element.

While optogenetic activation is suitable for studying acute state transitions (with millisecond-timescale precision), chemogenetic activation is better suited for studying long-term (minutes to hours) changes in sleep-wake behavior, LMA, and Tb. Therefore, we activated Nts^LH^ neurons using chemogenetic tools and assessed changes in sleep-wake, LMA, and Tb. We stereotaxically injected either an AAV vector (AAV-hM3Dq) coding for the excitatory designer receptor exclusively activated by designer drugs (DREADD) [15, 37] or a control vector (AAV8-DIO-hsyn-mCherry; henceforth “AAV-mCherry”) into the LH of Nts-Cre mice (Fig 5A) and implanted them with telemetry transmitters for simultaneous recordings of EEG, EMG, Tb, and LMA [15].

Similar to the optogenetic experiments, the AAV injections induced specific expression of the viral vector product in Nts^LH^ neurons, which was primarily restricted to the LH (Fig 5B). Bath application of 5 μM clozapine-n-oxide ([CNO] the ligand for hM3Dq receptors) depolarized hM3Dq-mCherry–expressing Nts^LH^ neurons and increased their firing rate in ex vivo brain slices (Fig 5C). Moreover, intraperitoneal (i.p.) injections of CNO (0.3 mg/kg) 2.5 h prior to killing induced robust cFos expression in hM3Dq-mCherry–expressing Nts^LH^ neurons (93.76% ± 1.52% of mCherry+ neurons expressed cFos after CNO in AAV-hM3Dq–injected Nts-Cre mice versus 16.78% ± 4.45% in AAV-mCherry–injected controls; Fig 5D). These data demonstrated that CNO effectively activated hM3Dq-expressing Nts^LH^ neurons both ex vivo and in vivo.

To assess sleep-wake, LMA, and Tb changes following chemogenetic activation of Nts^LH^ neurons, we i.p. injected saline (vehicle) or CNO (0.3 mg/kg) at 9:50 AM (light period) or 6:50 PM (dark period) and recorded EEG, EMG, LMA, and Tb for 12 h using the telemetry transmitters. Administration of CNO into Nts-Cre mice injected with the control vector AAV-mCherry (negative controls) did not produce any significant changes in sleep-wake, Tb, or LMA (data can be found from the Open Science Framework, https://osf.io/nmrpq/). Conversely, in the Nts-Cre mice expressing hM3Dq, CNO injections during the light period (9:50 AM) resulted in prolonged and uninterrupted wake (without any NREM or REM sleep) for 4–6 h (Fig 5E and S1 Table). The first NREM sleep and REM sleep were observed 275.00 ± 33.00 min and 312.66 ± 38.31 min, respectively, after CNO injections, significantly later than in the saline condition (40.74 ± 4.11 min and 68.29 ± 6.40 min, respectively; Fig 5F). Towards the end of the 12-h recording period, specifically hours 10–12 after CNO, we observed that REM sleep amounts, bout number, and mean duration were significantly higher than those after saline treatment, indicating a REM sleep rebound (Fig 5E and S1 Table). Although the NREM sleep increase during this same period did not reach statistical significance, a trend was observed, along with a significant increase in the number of NREM sleep bouts, indicating rebound increase in NREM sleep as well (Fig 5E and S1 Table). Finally, CNO injections at the dark onset (6:50 PM), when the circadian drive for wake is high, produced similar sleep-wake changes in Nts-Cre mice (S1 Fig and S2 Table).

The increase in wakefulness after CNO was accompanied by significant increases in Tb and LMA during both light (Tb: 1.56 ± 0.18°C and LMA: 584.81% ± 98.92% increase) and dark periods (Tb: 0.85 ± 0.09°C; LMA: 269.18% ± 22.45% increase) (Fig 5G and 5H and S1 Fig). Hyperthermia and higher LMA after CNO during the dark period were particularly interesting as their levels were much higher than the usual circadian increase in LMA and Tb at that time of day. Moreover, LMA per unit time of wake was significantly elevated after CNO (light period: 3.01 ± 0.50 versus 1.11 ± 0.13 counts per min after saline; *P* = 0.0084; dark period: 3.29 ± 0.61 versus 1.46 ± 0.19 counts per min after saline; *P* = 0.039), indicating a hyperactive phenotype. These data suggest that Nts^LH^ neurons directly increase LMA and Tb in addition to regulating sleep-wake behavior.

Thus, chemoactivation of Nts^LH^ neurons produced robust wake, hyperactivity, and hyperthermia, which are commonly observed after acute stress in rodents. While hyperthermia itself is considered a sensitive marker of stress [38], we also counted the cFos+ neurons in the paraventricular nucleus (PVH) whose activation in response to stressors leads to corticosterone secretion) [39, 40] as an additional stress marker. CNO injections almost doubled the cFos+ neurons in the hM3Dq-injected Nts-Cre mice when compared with negative controls (197.13 ± 32.57 versus 103.67 ± 18.01; *P* < 0.05, Mann–Whitney *U* test; S2 Fig), suggesting that activation of PVH occurs concurrently with the activation of Nts^LH^ neurons.

### Experiment 6: Chemoinhibition of Nts^LH^ neurons does not alter spontaneous sleep-wake behavior

**Fig 6:**
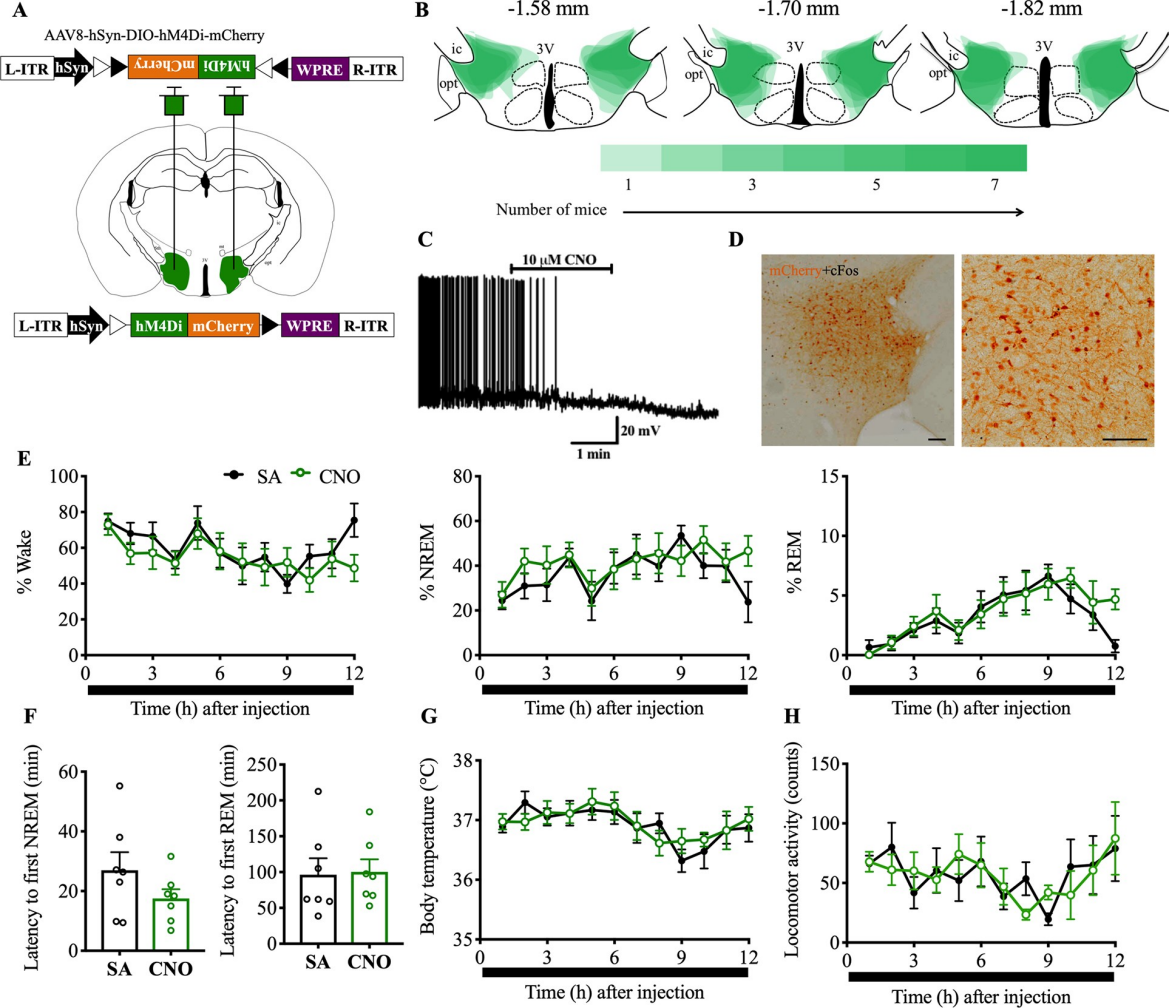
Chemoinhibition of Nts^LH^ neurons does not alter sleep-wake states or Tb during baseline conditions. (A) Schematic representation of AAV-hM4Di injections. (**B**) Heat map of AAV-hM4Di injection sites in the LH at three levels (anteroposterior: −1.58, −1.7, and −1.82 mm [36]); darker shades of green indicate more overlap of injection sites of different mice. (**C**) Ex vivo bath application of CNO (10 μM) hyperpolarized and inhibited the mCherry-expressing Nts^LH^ neurons (*n* = 4) in brain slices. (**D**) Robust reduction (near-complete absence) in cFos expression (black nuclei) after CNO (1.5 mg/kg; i.p) in hM4D4i-mCherry–expressing neurons in the LH (brown). Hourly percentages of different sleep-wake states (**E**), mean Tb (**G**), and total LMA counts (**H**) for 12 h after saline or CNO (1.5 mg/kg) administration at 6:50 PM in Nts-Cre mice injected with AAV-hM4Di into the LH. Two-way RM ANOVA for “time” and “compound injected,” followed by Sidak post hoc test (*n* = 7 mice; for wake: interaction *F*(11, 72) = 1.38, *P* = 0.20, compound injected *F*(11, 72) = 1.59, *P* = 0.12, time *F*(1, 72) = 5.08, *P* = 0.027; for NREM: interaction *F*(11, 72) = 1.40, *P* = 0.19, compound injected *F*(11, 72) = 1.29, *P* = 0.25, time *F*(1, 72) = 5.41, *P* = 0.023; for REM: interaction *F*(11, 72) = 0.93, *P* = 0.52, compound injected *F*(11, 72) = 4.29, *P* < 0.0001, time *F*(1, 72) = 1.46, *P* = 0.23; for Tb: interaction *F*(11, 72) = 0.54, *P* = 0.54, compound injected *F*(11, 72) = 1.82, *P* = 0.067, time *F*(1, 72) = 0.38, *P* = 0.54; for LMA: interaction *F*(11, 72) = 1.10, *P* = 0.37, compound injected *F*(11, 72) = 0.95, *P* = 0.50, time *F*(1, 72) = 0.015, *P* = 0.90). Latency to NREM and REM sleep (**F**) after saline and CNO injections. Mann–Whitney test (*n* = 7 mice: for NREM: *P* = 0.26; for REM: *P* = 0.62). Data are mean ± SEM. *P < 0.05, **P < 0.01. The underlying data for this figure are available from the Open Science Framework (https://osf.io/nmrpq/). CNO, clozapine-n-oxide; hSyn, human Synapsin-1; L-ITR, left inverted terminal repeat; LH, lateral hypothalamic area; LMA, locomotor activity; NREM, non-rapid eye movement; Nts, neurotensin; R-ITR, right inverted terminal repeat; REM, rapid eye movement; RM, repeated measures; SA, saline; Tb, body temperature; WPRE, woodchuck hepatitis virus posttranscriptional control element.

Next, we chemogenetically inhibited Nts^LH^ neurons to determine whether these neurons are necessary for spontaneous wake and regulation of Tb under baseline conditions. We stereotaxically injected an AAV encoding inhibitory DREADDs (AAV8-DIO-hsyn-hM4Di-mCherry; hereafter “AAV-hM4Di”) [34] into the LH of Nts-Cre mice (*n* = 7) (Fig 6A). Similar to experiment 5, we first confirmed the specific expression of hM4Di in Nts^LH^ neurons by immunohistologically staining brain slices 4 wk after viral injections (Fig 6B). Next, we assessed the response of hM4Di-transfected Nts^LH^ neurons to CNO in ex vivo brain slices and found that bath application of CNO (10 μM) caused complete inhibition of hM4Di-mCherry–expressing neurons (Fig 6C). Similarly, in vivo application of CNO (1.5 mg/kg, i.p.) in Nts-Cre mice 2.5 h before perfusions resulted in a complete absence of cFos in hM4Di-mCherry–expressing Nts^LH^ neurons (2.86% ± 1.52% of mCherry+ neurons expressed cFos in AAV-hM4Di–injected Nts-Cre mice versus 16.78% ± 4.45% in AAV-mCherry–injected controls; Fig 6D).

Administration of CNO (1.5 mg/kg, i.p.) during the light or dark period did not cause any major alterations in sleep-wake states in Nts-Cre mice expressing hM4Di in Nts^LH^ neurons (Fig 6E and S3 Fig). Hourly percentages, bout numbers, and bout durations of wake, NREM and REM sleep, as well as latencies to NREM and REM sleep after CNO were not significantly different from those after saline injections (Fig 6E and 6F, S3 Fig, S3 Table, and S4 Table). Similarly, total LMA and mean Tb after CNO were not significantly different from those after saline (Fig 6G and 6H and S3 Fig). These data indicate that Nts^LH^ neurons may not be critical for sleep-wake regulation or maintenance of Tb under baseline conditions.

### Experiment 7: Chemoinhibition of Nts^LH^ neurons attenuates responses to novelty stress but augments the responses to metabolic stress

Although the inhibition of Nts^LH^ neurons did not alter sleep-wake, LMA, and Tb during baseline conditions, activation of Nts^LH^ neurons produced robust wake, hyperactivity, and hyperthermia as well as increased cFos expression in the PVH—all of which are usually observed in response to stress [31]. We therefore hypothesized that Nts^LH^ neurons may specifically engage in stress-induced behavioral and physiological arousal. To test this hypothesis, we chemogenetically inhibited Nts^LH^ neurons and assessed sleep-wake, LMA, and Tb in response to (a) the psychological stress induced by a novel environment and (b) the physiological/metabolic stress induced by fasting.

**Fig 7:**
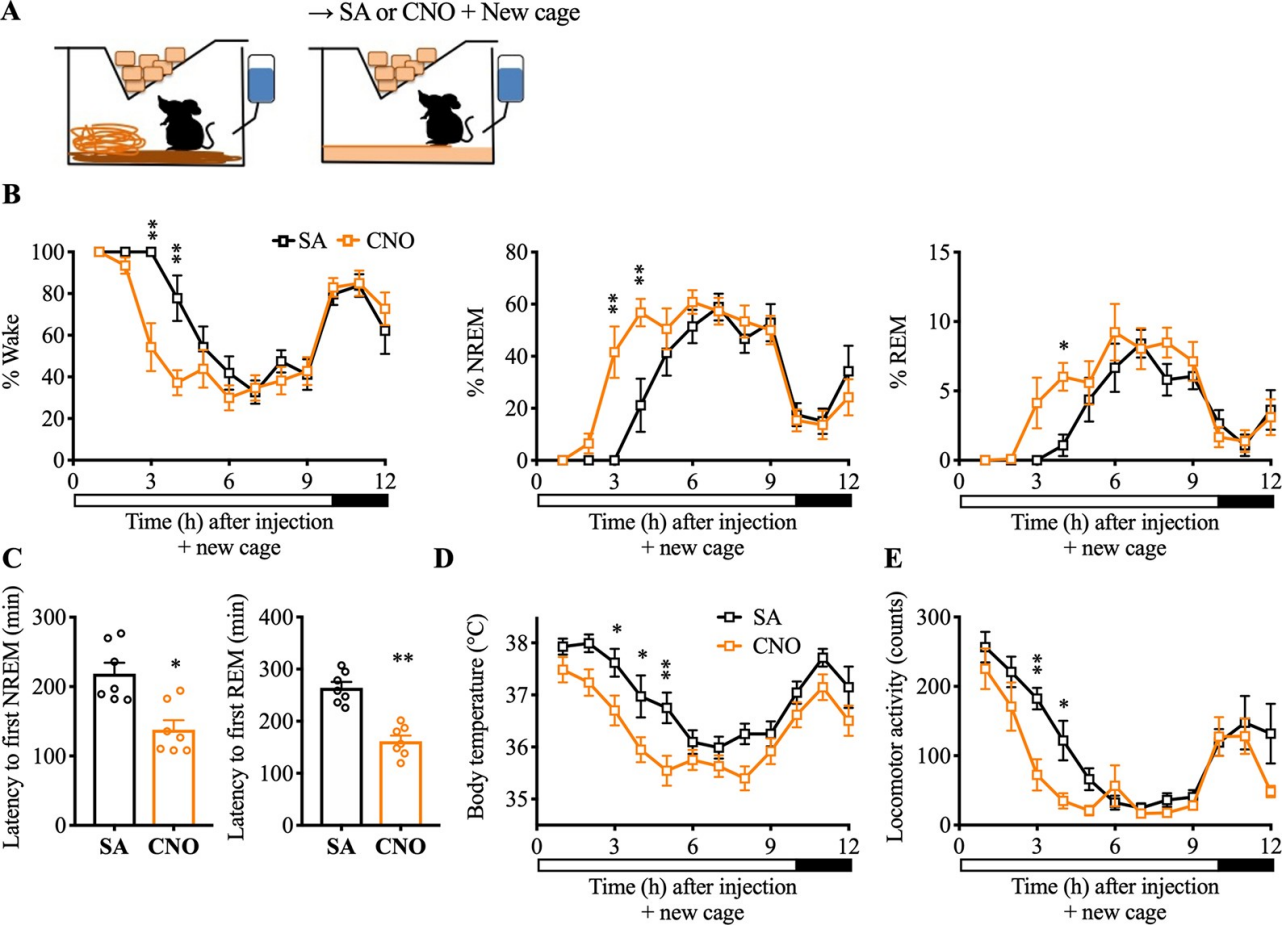
Chemoinhibition of Nts^LH^ neurons attenuates stress-induced arousal and hyperthermia. (**A**) Schematic of novel environment experimental setup. Mice were introduced to a new cage with fresh nesting material immediately after i.p. injections of saline or CNO at 9:50 AM. Hourly percentages of sleep-wake stages (**B**), mean Tb (**D**), and total LMA counts (**E**) for 12 h after saline or CNO (1.5 mg/kg) administration and cage change. Two-way RM ANOVA for the first 12 h after treatment for “time” and “compound injected,” followed by Sidak post hoc test (*n* = 7 mice; for wake: interaction *F*(11, 72) = 3.20, *P* = 0.0014, compound injected *F*(11, 72) = 24.15, *P* < 0.0001, time *F*(1, 72) = 9.97, *P* = 0.0023; for NREM: interaction *F*(11, 72) = 3.20, *P* = 0.0014, compound injected *F*(11, 72) = 23.37, *P* < 0.0001, time *F*(1, 72) = 9.39, *P* = 0.0031; for Tb: time *F*(1, 72) = 55.44, *P* < 0.0001, compound injected *F*(11, 72) = 12.13, *P* < 0.0001; for REM: interaction *F*(11, 72) = 1.43, *P* = 0.18, compound injected *F*(11, 72) = 12.82, *P* < 0.0001, time *F*(1, 72) = 7.45, *P* = 0.0080; for Tb: interaction *F*(11, 72) = 0.93, *P* = 0.51, compound injected *F*(11, 72) = 12.13, *P* < 0.0001, time *F*(1, 72) = 55.44, *P* < 0.0001; for LMA: interaction *F*(11, 72) = 1.92, *P* = 0.051, compound injected *F*(11, 72) = 18.43, *P* < 0.0001, time *F*(1, 72) = 18.12, *P* < 0.0001). (**C**) NREM and REM sleep latencies after saline or CNO injections and cage change: Mann–Whitney test (*n* = 7 mice; for NREM: *P* = 0.011; for REM: *P* = 0.0006). Data are mean ± SEM. *P < 0.05, **P < 0.01. When placed into the new cage during their rest period, mice display increased wake, hyperactivity, and hyperthermia for 3–4 h before they go to sleep. Chemoinhibition of Nts^LH^ neurons attenuates these responses to the novel environment stress. The underlying data for this figure are available from the Open Science Framework (https://osf.io/nmrpq/). CNO, clozapine-n-oxide; i.p., intraperitoneal; LH, lateral hypothalamic area; LMA, locomotor activity; NREM, non-rapid eye movement; Nts, neurotensin; REM, rapid eye movement; RM, repeated measures; SA, saline; Tb, body temperature.

To study the role of Nts^LH^ neurons in the stress response induced by a novel environment, we injected mice expressing hM4Di in Nts^LH^ neurons with CNO (1.5 mg/kg, i.p.) or saline at 9:50 AM and immediately placed them in a new, clean cage with fresh bedding material (Fig 7A). Mice placed in a new cage after saline injections (saline+new cage) showed a significant increase in wake, LMA, and Tb for approximately 4 h compared with saline injections in the home cage (Fig 7B–7E and S5 Table). The first NREM sleep and REM sleep bouts in the new cage were observed after 218.74 ± 15.71 min and 264.03 ± 11.51 min, respectively (Fig 7C). In contrast, when the mice were injected with CNO before placing them in a new cage (CNO+new cage), sleep onset occurred faster with significantly decreased latencies for both NREM sleep (137.89 ± 13.65 min) and REM sleep (161.60 ± 11.05 min) (Fig 7C). Consistently, the percentage of time spent in NREM and REM sleep in the third hour (NREM: 41.57% ± 9.90% versus 0.00% ± 0.00% after saline; *P* < 0.0001; REM: 4.13% ± 1.83% versus 0.00% ± 0.00% after saline; *P* = 0.13) and the fourth hour (NREM: 56.74% ± 5.29% versus 21.14% ± 10.22% after saline, *P* = 0.0007; REM: 6.01% ± 1.01% versus 1.09% ± 0.77% after saline, *P* = 0.034) after CNO+new cage were higher than in the saline+new cage condition (Fig 7B). Similarly, the LMA and Tb during the period of 3–5 h after CNO+new cage were also substantially lower than after saline+new cage (Fig 7D and 7E). These findings demonstrate that Nts^LH^ neurons are necessary for modulating arousal, LMA, and hyperthermia in response to psychological stress induced by a novel environment.

**Fig 8:**
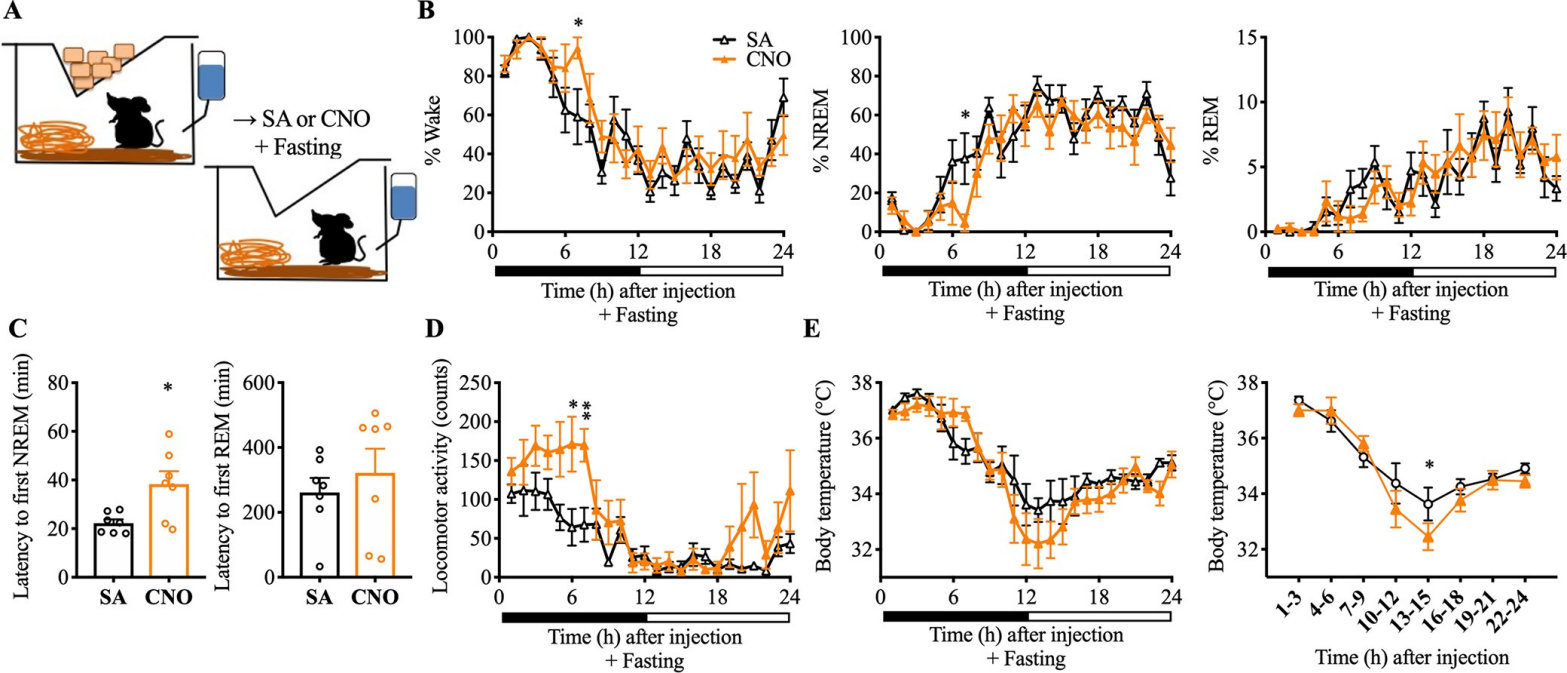
Chemoinhibition of Nts^LH^ neurons amplifies arousal responses to a metabolic stress. (**A**) Schematic of metabolic stress (fasting) experiment setup. Mice were injected with saline or CNO (1.5 mg/kg) i.p. and fasted for the next 24 h. Food was completely removed from the cage after the injections. Hourly percentages of sleep-wake stages (**B**), total LMA counts (**D**), and mean Tb (**E**) for 24 h after saline or CNO (1.5 mg/kg) administration and fasting. Mean Tb in 3-h intervals are also provided to highlight the hypothermia observed after CNO+fasting. Two-way RM ANOVA for 24 h after treatment for “time” and “compound injected,” followed by Sidak post hoc test (*n* = 7 mice; for wake: interaction *F*(23, 144) = 1.23, *P* < 0.23, compound injected *F*(23, 144) = 13.76, *P* < 0.0001, time *F*(1, 144) = 4.47, *P* = 0.036; for REM: interaction *F*(23, 144) = 0.66, *P* = 0.88, compound injected *F*(23, 144) = 7.13, *P* < 0.0001, time *F*(1, 144) = 0.029, *P* = 0.86; for NREM: interaction *F*(23, 144) = 1.24, *P* = 0.22, compound injected *F*(23, 144) = 13.54, *P* < 0.0001, time *F*(1, 144) = 5.33, *P* = 0.022; for Tb: interaction *F*(23, 144) = 1.24, *P* = 0.22, compound injected *F*(23, 144) = 12.34, *P* < 0.0001, time *F*(1, 144) = 7.86, *P* < 0.0057; for LMA: interaction *F*(23, 144) = 1.54, *P* = 0.066, compound injected *F*(23, 144) = 7.58, *P* < 0.0001, time *F*(1, 144) = 30.63, *P* < 0.0001). (**C**) NREM and REM sleep latencies after saline or CNO injections and food deprivation: Mann–Whitney test (*n* = 7 mice; for NREM: *P* = 0.038; for REM: *P* = 0.38). Data are mean ± SEM. *P < 0.05, **P < 0.01. When fasted during their active (feeding) period, mice exhibit increased wake, hyperactivity, and hyperthermia initially (food foraging), which was followed by a period of increased sleep and hypothermia (about 15 h after). Chemoinhibition of Nts^LH^ neurons amplified both the early and late responses to fasting. The underlying data for this figure are available from the Open Science Framework (https://osf.io/nmrpq/). CNO, clozapine-n-oxide; i.p., intraperitoneal; LH, lateral hypothalamic area; LMA, locomotor activity; NREM, non-rapid eye movement; Nts, neurotensin; REM, rapid eye movement; RM, repeated measures; SA, saline; Tb, body temperature.

Based on the established role of the LH in feeding and energy homeostasis [41, 42], we then hypothesized that Nts^LH^ neurons might contribute to the regulation of sleep-wake, LMA, and Tb in response to acute fasting (or mealtime hunger)—a form of physiologic/metabolic stress. Therefore, we tested whether Nts^LH^ neurons contribute to these responses after metabolic stress by inhibiting hM4Di-expressing Nts^LH^ neurons at dark onset by i.p. injections of CNO in the absence of food (Fig 8, S4 Fig, and S6 Table). Food was removed during the same time as i.p. injections and mice were not habituated to fasting or food restriction prior to these experiments. We recorded sleep-wake behavior, LMA and Tb during the 24-h fasting period following CNO.

When fasted after saline injections (saline+fasting), Nts-Cre mice (expressing hM4Di in Nts^LH^ neurons) displayed an increase in wakefulness, with a corresponding decrease in NREM sleep for 3 h (between 2 and 4 h after injections; S4 Fig). This wake increase was accompanied by an increase in LMA (55% higher than in the saline+fed condition). Following this period (i.e., 4 h after fasting), both Tb and LMA began to drop (as expected in fasting mice), and we observed a significant increase in hypothermia (<34°C) and hypoactivity between 11 and 16 h after saline+fasting (S4 Fig). Similarly, NREM sleep during the same period was higher than in the saline+fed condition (S4 Fig).

Interestingly, chemoinhibition of Nts^LH^ neurons exaggerated the sleep-wake and Tb responses to fasting. When fasted after CNO injections (CNO+fasting), Nts-Cre mice expressing hM4Di in Nts^LH^ neurons displayed increased wakefulness for 6–7 h (Fig 8B). While the amount of wakefulness during the first 3 h after CNO+fasting was comparable to that in the saline+fasting condition, it was significantly higher during the seventh hour after CNO+fasting (94.47 ± 5.41% versus 59.10 ± 14.19% after saline; *P* = 0.044). Importantly, the latency to NREM sleep was significantly longer after CNO+fasting compared with saline+fasting (Fig 8C). LMA levels remained elevated for 6–7 h after CNO+fasting compared with an increase lasting only 3 h after saline+fasting (Fig 8D and S4 Fig). Similarly, Tb levels started falling 7 h after CNO+fasting (compared with 5 h after saline), but fell more rapidly and with a greater magnitude between 13 and 15 h after CNO+fasting (versus saline+fasting; Fig 8E); this later response may not be a direct consequence of the inhibition of Nts^LH^ neurons, because the plasma half-life of CNO is short (<1 h) and its behavioral effects generally last about 4 to 8 h [43–45]. In contrast, this deeper fall in Tb between 13 and 15 h is presumably a consequence of enhanced wake and hyperactivity during the first 6–7 h after CNO. These results indicate that Nts^LH^ neurons are critical for the fine-tuning of sleep-wake behavior, LMA, and Tb in times of caloric scarcity. Naturally, when food is unavailable, mice must reduce their activity and levels of wakefulness to conserve energy and guard against metabolic stress. Our findings suggest that Nts^LH^ neurons are critical for this response.

## Discussion

Our results establish Nts^LH^ neurons as a distinct neuronal population in the LH without overlap with either the orexin or MCH population. Using optogenetic and chemogenetic approaches, we demonstrate that (1) acute activation of Nts^LH^ neurons results in rapid arousals from NREM sleep but not from REM sleep; (2) sustained activation of Nts^LH^ neurons causes prolonged wake, hyperactivity, and hyperthermia; and (3) inhibition of Nts^LH^ neurons attenuates the arousal and hyperthermia response to psychological stress, but augments sleep-wake, LMA, and Tb responses to metabolic stress.

Our data indicate that Nts^LH^ neurons are distinct from orexin and MCH neurons in the LH, which is consistent with a previous report [46] but contradicts another study that reported a >80% overlap between of Nts^LH^ and orexin neurons [26]. It is likely that the specificity of the antibodies and mRNA probes used by Furutani and colleagues prevented them from distinguishing between signals from Nts^LH^ cell bodies and dense Nts terminals on orexin neurons [26]. While we observed a small fraction of Nts neurons expressing orexin in the Nts-GFP reporter mice, no such colocalization was observed when Nts neurons were visualized in adult mice. Thus, it appears that some Nts^LH^ neurons may be capable of synthesizing orexin during development, but not in the adult stage. Consistently, single-cell gene expression analysis revealed no evidence of Nts mRNA in orexin neurons in adult mice [47]. These observations establish Nts^LH^ as a distinct population of LH neurons in adult mice. While Nts^LH^ neurons do not express MCH or orexin, subpopulations of Nts^LH^ neurons may co-express other neuropeptides, including galanin and corticotrophin-releasing hormone, and classical neurotransmitters such as GABA and glutamate [20, 48–50].

### Nts^LH^ neurons in sleep-wake regulation and Tb control

We show that brief optogenetic activation of Nts^LH^ neurons produces immediate transitions to wake from NREM sleep, while sustained chemogenetic activation causes arousals lasting several hours, suggesting that Nts^LH^ neurons play a crucial role in both initiation and maintenance of wakefulness. While rapid arousals from NREM sleep were consistently evoked by photoactivation of Nts^LH^ neurons, arousals from REM sleep were never evoked. Because even high-frequency photostimulations failed to arouse mice from REM sleep, it is not likely that differences in arousal threshold between NREM sleep and REM sleep are responsible. On the other hand, it is possible that Nts^LH^ neurons become unresponsive to external stimuli during REM sleep, which is an inherent property of hypothalamic thermosensitive neurons [51, 52]. Considering the hyperthermia induced by Nts^LH^ neuronal activation, these neurons may belong to a cold-sensitive neuronal population and may engage in cold defense behavior.

Another possibility is that the arousal from REM sleep involves pathways and mechanisms different from those involved in the arousal from NREM sleep and that Nts^LH^ neurons may be a part of the latter. Interestingly, the previously identified wake-promoting cell groups in the forebrain, such as GABAergic neurons in the LH [11], bed nucleus of stria terminalis (BNST) [53], and basal forebrain [54–56], also did not elicit wakefulness from REM sleep. Considering the presence of sleep-wake alternation without REM sleep in the isolated forebrain [57, 58], it is probable that most wake-promoting neurons in the forebrain are wired for arousal from NREM sleep but not from REM sleep.

Besides the rapid but relatively short arousals induced by optogenetic stimulation of Nts^LH^ neurons, we show that arousals induced by chemogenetic activation of Nts^LH^ neurons were long-lasting and accompanied by a hyperactivity and hyperthermia, suggesting that the Nts^LH^ neurons may regulate LMA and Tb in addition to sleep-wake states. Although wake is associated with increased activity, the LMA count per unit time of wake was significantly higher after Nts^LH^ activation than during baseline wake, suggesting mice were hyperactive. Wake and LMA do not always go hand in hand. For example, increased wake after systemic administration of certain pharmacological agents (e.g., modafinil) or after activation of the wake-promoting cell groups in the brain (e.g., PB) were not accompanied by increased LMA [59, 60]. On the contrary, increased LMA after loss of MCH neurons in the LH was not accompanied by an increase in wake [15]. Thus, the increased LMA after Nts^LH^ activation is not necessarily due to higher wake amounts.

Similarly, subchronic (30-min) photoactivation of Nts^LH^ caused an increase in Tb that was neither related to state changes nor accompanied by an increase in LMA, even though chemoactivation increased Tb with a concurrent increase in LMA. While chemoactivation makes neurons more responsive to natural inputs, photoactivation causes rhythmic monotonous firing of neurons. Such differential activation of Nts^LH^ neurons could have led to different downstream responses, contributing to the differential locomotor responses after optogenetic- versus chemoactivation. Nevertheless, these data clearly demonstrate that Nts^LH^ activation may increase Tb independent of wake or hyperactivity and thereby suggest a direct role for Nts^LH^ neurons in thermogenesis. Lack of arousal response after activation of Nts^LH^ neurons during REM sleep indicates the potential cold-sensitive nature of these neurons [51, 52], further supporting this idea. Future studies are, however, necessary to test the cold sensitivity of Nts^LH^ neurons and the thermoregulatory deficits induced by their loss.

### Nts^LH^ neurons are necessary for stress responses

In contrast to chemogenetic activation, chemogenetic inhibition of Nts^LH^ neurons had no effect on wake, LMA, or Tb, suggesting that these neurons may not be necessary for the regulation of spontaneous wakefulness or Tb control under baseline conditions. Because the increased wake, hyperthermia, and hyperactivity after chemogenetic activation of Nts^LH^ neurons resembled a stress response, with concurrent increase in cFos in PVH neurons, we hypothesized that these neurons could be important for stress-induced arousals. We found that chemoinhibition of Nts^LH^ neurons indeed attenuates wakefulness, LMA, and Tb responses to stress induced by a novel environment. Interestingly, Nts^LH^ inhibition paradoxically amplified the sleep-wake, LMA, and Tb responses to metabolic stress induced by fasting. Clearly, the responses to stress should differ depending on the type, magnitude, and duration of the stress [61]—while it may be necessary to increase wake to explore a novel environment and search for potential threats and food sources, it is also necessary to decrease wakefulness and reduce energy expenditure during metabolic challenges, such as during prolonged absence of food. Thus, the observed changes in response to novelty stress and metabolic stress are not actually paradoxical but instead indicate that Nts^LH^ neurons might integrate stress stimuli and generate the appropriate responses. It is also likely that Nts^LH^ neurons are heterogenous (in terms of co-expression of other neurotransmitter or molecular signatures) [20], and these different subsets of neurons may orchestrate responses to different stressors through their differential output pathways. Further studies are required to identify these subsets of Nts^LH^ neurons selectively responding to different stressors.

Similar to chemoinhibition of Nts^LH^ neurons, attenuated arousal response to a novelty stress was observed in orexin neuron-ablated mice, and exaggerated arousal response to fasting was observed in MCH-knockout mice [62, 63]. In addition, orexin neuron-ablated mice also did not exhibit increased arousal levels during fasting [63]. Both orexin and MCH neurons express receptors for Nts [22, 64], and both receive inputs from Nts^LH^ neurons. Importantly, Nts^LH^ neurons are the only cell population in the LH that expresses MCH receptors [65]. Nts has been shown to activate orexin neurons in vivo and ex vivo [26]. In contrast, activation of Nts terminals on orexin neurons may inhibit orexin neurons by releasing galanin [66]. Thus, it is possible that Nts neurons may excite or inhibit orexin neurons by releasing either Nts or galanin, respectively. Notably, Nts antagonists have no effect on sleep-wake states in orexin-ablated mice [26]. Thus, we propose that Nts^LH^ neurons may act as a “master orchestrator” and modulate the activity of orexin and MCH neurons, depending upon the perceived stressors, and generate appropriate stress responses.

Because we did not study the specific inputs to Nts^LH^ neurons, it is unclear how stress signals reach Nts^LH^ neurons. However, previous studies have shown that several major mediators of stress responses such as the medial prefrontal cortex, PVH, BNST, and amygdala heavily project to the LH [67–69], and Nts^LH^ neurons may receive these inputs. On the other hand, metabolic signals may directly target Nts^LH^ because a subset of these neurons expresses leptin receptors and Nts^LH^ neurons have been shown to be activated by leptin in brain slices [22, 70].

### Potential pathways underlying sleep-wake and Tb responses after Nts^LH^ activation

In addition to local LH circuits linking Nts neurons with orexin and MCH neurons, we observed direct projections from Nts neurons to other brain structures regulating sleep-wake, Tb, and LMA. Based on our tracing data, we predict that Nts^LH^ could activate the VTA, LC, and PB, which are known as potent wake-promoting cell groups [27, 71–74]. Likewise, Nts^LH^ neurons could inhibit the lateral preoptic area, which is sleep promoting [75, 76]. Moreover, activity in Nts^LH^ neurons could increase Tb by activating RPa/Ppy and ventromedial medulla neurons and promote nonshivering and shivering thermogenesis, respectively [77–80]. Finally, Nts^LH^ projections to the VTA may mediate the LMA responses, as increased LMA after Nts^LH^ neurons were blocked by dopamine antagonists, and intra-VTA administration of Nts-antagonist blocked the dopamine release from VTA neurons [81]. While Nts can be excitatory or inhibitory depending upon the receptor expression in the postsynaptic neurons, a subset of Nts^LH^ neurons also express GABA [81]. Thus, the hyperthermia, hyperactivity, and wakefulness after Nts^LH^ activation could be due to a complex integration of inhibitory and excitatory signals. Future studies are required to identify the specific neurotransmitter and pathways involved in each of these responses.

Collectively, our results indicate that Nts^LH^ neurons are capable of initiating and sustaining wakefulness and increasing Tb and LMA. While Nts^LH^ neurons may not be necessary for spontaneous wakefulness or Tb maintenance under baseline conditions, they are necessary for modulating wake and hyperthermia after psychological or metabolic stressors. We show that Nts^LH^ neurons reduce wake, LMA, and Tb in response to fasting, while they increase wake, LMA, and Tb in response to a novel environment. Moreover, Nts^LH^ neurons may also be cold sensitive and potentially contribute to cold defense mechanisms, as they were unresponsive during REM sleep, and their activation induced strong hyperthermia. Considering the involvement of the LH in various physiological functions, including sleep-wake, feeding, and thermoregulation and the close-interrelationship between these functions, [1–3, 20, 82–85], our results suggest that Nts^LH^ neurons may play a crucial role in modulating sleep-wake states, LMA, and Tb in response to a variety of physiologic and metabolic demands.

## Methods

### Ethics statement

All experiments were conducted in accordance with the National Institutes of Health guidelines for the Care and Use of Laboratory Animals and were approved by the institutional animal care and use committee of Beth Israel Deaconess Medical Center (protocol #039–2016). All efforts were made to minimize the number of animals used and their suffering.

### Animals

Prior to surgery, all mice were group-housed in a temperature (22 ± 1°C)–and humidity (40%–60%)–controlled animal room maintained on a 12:12-h light-dark cycle. All mice had ad libitum access to standard chow diet and water. After surgery, all animals were singly housed for 3–4 wk before the physiological data collection began. Male mice aged 8–12 wk and weighing between 20 and 24 g at the time of surgery were used for behavioral experiments, and 4-wk-old mice were used for ex vivo brain slice recordings. For this study, we used two transgenic mouse lines—mice expressing Cre recombinase under the Nts promoter (Nts^tm1(cre) Mgmi^/J mice; Jackson Laboratory, Stock No. 017525; “Nts-Cre mice” [22]) and a GFP-reporter mouse line (Rosa26-loxSTOPlox-L10-GFP; generated by Dr. Brad Lowell, BIDMC; “L10-GFP mice” [86]). Nts-Cre mice were crossed with L10-GFP mice to validate Cre expression in Nts neurons. For all experiments, we used heterozygous Nts-Cre mice on a mixed background.

### Genotyping

Genomic DNA from mice was extracted from tail biopsies and analyzed via polymerase chain reaction using a REDE Extract-N-Amp Tissue PCR Kit (Sigma-Aldrich, US) (Nts-Cre: common forward, 5′-ATA GGC TGC TGA ACC AGG AA; WT reverse, 5′-CAA TCA CAA TCA CAG GTC AAG AA; Cre reverse, 5′-CCA AAA GAC GGC AAT ATG GT. Rosa26-loxSTOPlox-L10-GFP: WT forward, 5′-GAG GGG AGT GTT GCA ATA CC; mutant forward, 5′-TCT ACA AAT GTG GTA GAT CCA GGC; and common reverse, 5′-CAG ATG ACT ACC TAT CCT CCC).

### Surgery for anatomical characterization and tracing experiments (Experiments 1 and 2)

Adult male Nts-Cre mice were anesthetized with a ketamine/xylazine mixture (100 mg/kg ketamine and 10 mg/kg xylazine) and were unilaterally microinjected with 60 nL of AAV-ChR2 (University of North Carolina Vector core) or AAV-hM3Dq (University of North Carolina Vector core) into the LH (anteroposterior, −1.7 mm from bregma; lateral ±1.1 mm; dorsoventral, −5.1 mm from dura [36]). Six weeks after the injections, all mice were killed for histological processing.

### Surgery and recordings for optogenetic experiments (Experiments 3 and 4)

Adult male Nts-Cre mice were anesthetized with a ketamine/xylazine mixture (100 mg/kg ketamine and 10 mg/kg xylazine) and were microinjected bilaterally with 60 nL AAV-ChR2 or AAV8-EF1α-DIO-mCherry (AAV-mCherry; University of North Carolina Vector core) into the LH [15]. All mice were then implanted with (a) optical fibers targeting 0.2 mm dorsal to the LH for blue light/laser stimulation, (b) electrodes for recording EEG and EMG-EEG signals by using ipsilateral stainless steel screws and EMG signals by a pair of stainless steel wires inserted into the neck extensor muscles, and (c) i.p. radio transmitter (TA10TA-F20, Data Science International, MN) for measuring Tb and LMA [15, 34].

Four weeks after surgery and AAV microinjections, mice were connected to the recording cables and habituated for 3 d, after which we performed baseline sleep-wake, LMA, and Tb recordings. The EEG/EMG signals were amplified (AM systems, WA, US), digitized, and recorded using Vital recorder (Kissei Comtec, Nagano, Japan) [75]. Tb and LMA data were recorded using Dataquest ART 4.1 (Data Sciences International, MN). We examined the effects of laser stimulation (10 s of stimulation at 1, 5, and 10 Hz) after 30 s of stable NREM sleep (10 trials each stimulation frequency) or 10 s of stable REM sleep (10 trials each stimulation frequency) during the light period. For assessing changes in LMA and Tb, we applied 5-Hz laser stimulation for 30 min during the light period.

### Surgery and recordings for chemogenetic experiments (Experiments 5, 6, and 7)

Nts-Cre mice were anesthetized with ketamine/xylazine (100 mg/kg and 10 mg/kg, i.p.) and injected with AAV8-hsyn-DIO-hM3Dq-mCherry, AV8-hsyn-DIO-hM4Di-mCherry, or AAV-mCherry bilaterally into the LH and were implanted with the telemetry transmitters (TL11M2-F20-EET; Data Science International, St. Paul, MN) that allow simultaneous recording of EEG, EMG, LMA, and Tb [15, 87]. Four weeks after surgery, mice were habituated to the recording room conditions for 3 d. For the interventions, mice were injected with the ligand for hm3Dq and hM4Di, CNO (0.3 or 1.5 mg/kg; Sigma, St. Louis, MO), or the vehicle (saline) at 9:50 AM (10 min before ZT3) or 6:50 PM (10 min before dark onset), and postinjection recordings of sleep-wake, LMA, and Tb (Dataquest ART 4.1, Data Sciences International, US) were performed for 24 h. The order of injections was counterbalanced and there were approximately 7 d between two CNO injections in the same mouse.

### Data analysis

The EEG, EMG data were divided into 12-s epochs and scored manually into one of the three sleep-wake states, wake, NREM sleep, or REM sleep, using SleepSign 3 (Kissei Comtec, Nagano, Japan) [15, 34, 75]. Percentages of time spent in each sleep-wake state and their mean number and bout durations in 1- and 3-h bins were calculated, along with the total LMA and mean Tb for these periods. Latency to NREM sleep and REM sleep were calculated as time taken to that stage from the time of i.p. injections.

### Histology

After the completion of physiological data collection, all mice were deeply anesthetized with 7% of chloral hydrate and were transcardially perfused with PBS (15 mL) followed by 10% formalin (50 mL). Mouse brains were harvested immediately and incubated with 10% formalin overnight, followed by incubation in 20% sucrose in formalin solution at 4°C. Brains were cut into three series of 40-μm coronal sections on a freezing microtome and processed for immunohistochemistry, immunofluorescence, and/or in situ hybridization.

For immunohistochemistry using diaminobenzidine (DAB) reactions, sections were incubated with the primary antibody for two nights (for cFos labeling) or overnight (all others), followed by incubation in the appropriate biotin-SP-conjugated secondary antibody (1:1,000; Jackson ImmunoResearch, West Grove, PA) for 2 h. Then, sections were incubated for 75 min in avidin-biotin-complex reagent (1:1,000; Vectastain ABC kit, Vector Lab, Burlingame), washed, and incubated in a 0.06% solution of DAB (Sigma-Aldrich) and 0.02% H2O2 for 2–5 min for staining in brown. CoCl2 (0.05%) and 0.01% NiSO4 (NH4) in PBS was added to the DAB solution for staining in black [15, 34, 87, 88].

For immunofluorescence, sections were incubated in primary antibodies overnight. After washes in PBS, the sections were incubated in the appropriate fluorescent secondary antibodies (1:1,000; Alexa Fluor Dyes, Life Technologies, Carlsbad, CA) for 2 h.

The following antibodies were used: primary antibody for cFos (1:20,000; PC38; MilliporeSigma, Darmstadt, Germany), ds-Red (1:10,000; 632496; Clontech Laboratory, Mountain View, CA), Orexin-A (1: 5,000; SC-8070; Santa Cluz Biotechnology, Dallas, TX), and MCH (1: 5,000; gift from Dr. Eleftheria Maratos-Filer, Harvard University, Boston, MA) [15, 34, 88, 89].

Sections were mounted on Superfrost glass slides, dehydrated, cleared, and coverslipped using Permaslip (Albose Scientific, MO) in case of DAB staining or Vectashield (Vector labs, CA) in case of fluorescent labeling.

### Cell counting

All cell counting was performed by constructing a 500 × 500 μm box on the lateral hypothalamus. The dorsal border of the square was aligned with the dorsal edge of the third ventricle, while the lateral border was aligned with the lateral edge of the hypothalamus [89]. The Franklin and Paxinos mouse brain atlas [36] was used for determining anteroposterior coordinates. Abercrombie corrections were applied to all cell counts [90].

### Ex vivo electrophysiological recordings

Under anesthesia, Nts-Cre mice (4 wk old) were injected with AAV-ChR2 (*n* = 3), AAV-hM3Dq (*n* = 4), or AAV-hM4Di (*n* = 3) into the LH and killed after 4 wk. Brains were removed and quickly transferred to ice-cold cutting solution consisting of 72 mM sucrose, 83 mM NaCl, 2.5 mM KCl, 1 mM NaH2PO4, 26 mM NaHCO3, 22 mM glucose, 5 mM MgCl2, and 1 mM CaCl2, carbogenated with 95% O2/5% CO2, with a measured osmolarity of 310–320 mOsm/L. Brains were cut into 250-μm slices and the slices containing the LH were used for current-clamp recordings. mCherry (ChR2/hM3Dq/hM4Di)–expressing Nts neurons in the slices were visualized using an upright microscope (SliceScope, Scientifica), and current-clamp recordings were performed using borosilicate glass microelectrodes (5–7 MΩ) filled with internal solution [33, 86, 91]. After achieving stable baseline recordings for 5–10 mins, the response to photo-illumination or CNO (depending upon the AAV injected) was investigated. To test the response of ChR2-expressing Nts^LH^ neurons to photo-illumination, 477 nm light was applied for 5–10 s at various frequencies (1–10 Hz), and the recordings were continued for 1–2 min. Data from 10 s before, during, and after the stimulus were compared. To test the CNO effects of hM3Dq- or hM4Di-expressing Nts^LH^ neurons, artificial cerebrospinal fluid (ACSF) solution containing CNO (500 nM) was perfused onto the slice preparation and recordings continued for 2–5 min, followed by ACSF perfusions to wash out the CNO. Data from 2 min just prior to bath application of CNO were considered as baseline; the response to CNO was measured during the last 1 min of CNO application. The resting membrane potentials before and during CNO were compared using paired *t* tests. Current (5–20 pA) was applied via the patch pipette if mCherry+neurons did not fire action potentials (for hM4Di inhibition experiments).

### Statistical analysis

Statistical analysis was performed using GraphPad Prism version 7 (GraphPad Software, La Jolla, CA). For optogenetic experiments, data after photostimulations (in Nts-Cre mice injected with AAV-ChR2) were compared with prestimulation data as well as after sham stimulations using a one-way ANOVA followed by Tukey multiple comparisons. In chemogenetic experiments, post-CNO data from the experimental group (Nts-Cre mice injected with AAV-hM3Dq/AAV-hM4Di) were compared with post-saline data from the same mice and post-CNO data from negative controls (Nts-Cre mice injected with AAV-mCherry) using a two-way repeated measures ANOVA, followed by Sidak post hoc test. All data are presented as the mean ± SEM unless otherwise noted. Differences were considered significant at *P* values less than 0.05.

## Supporting information

S1 Fig Chemoactivation of Nts^LH^ neurons during the dark period causes sustained arousals and hyperthermia. Hourly percentages of different sleep-wake states (**A**), mean Tb (**C**), and total LMA counts (**D**) for the 12 h after saline or CNO (0.3 mg/kg) administration at 06:50 PM in Nts-Cre mice injected with AAV-hM3Dq into the LH. Latency to NREM and REM sleep (**B**) after saline and CNO injections. Two-way RM ANOVA for “time” and “compound injected,” followed by Sidak post hoc test (*n* = 7 mice; for wake: interaction *F*(11,72) = 5.53, *P* < 0.0001, compound injected *F*(11, 72) = 15.45, *P* < 0.0001, time *F*(1, 72) = 17.54, *P* < 0.0001; for NREM: interaction *F*(11,72) = 5.84, *P* < 0.0001, compound injected *F*(11, 72) = 15.68, *P* < 0.0001, time *F*(1, 72) = 19.05, *P* < 0.0001; for REM: interaction *F*(11,72) = 2.00, *P* = 0.041, compound injected *F*(11, 72) = 4.64, *P* < 0.0001, time *F*(1, 72) = 3.96, *P* < 0.051; for Tb: interaction *F*(11,72) = 4.28, *P* < 0.0001, compound injected *F*(11, 72) = 6.05, *P* < 0.0001, time *F*(1, 72) = 21.64, *P* < 0.0001; for LMA: interaction *F*(11,72) = 6.76, *P* < 0.0001, compound injected *F*(11, 72) = 9.97, *P* < 0.0001, time *F*(1, 72) = 45.09, *P* < 0.0001). Data are mean ± SEM. *P < 0.05, **P < 0.01. The underlying data for this figure are available from the Open Science Framework (https://osf.io/nmrpq/). CNO, clozapine-n-oxide; LH, lateral hypothalamic area; LMA, locomotor activity; NREM, non-rapid eye movement; Nts, neurotensin; REM, rapid eye movement; RM, repeated measures; Tb, body temperature.
(TIFF)

S2 Fig Chemoactivation of Nts^LH^ neurons increases cFos in the paraventricular hypothalamus. Representative brain sections at the level of PVH (marked by dashed lines) labeled for cFos (black dots) from Nts-Cre mice injected with AAV-hM3Dq or AAV-mCherry into the LH (A). These mice were injected with CNO i.p. 2.5 h before they were killed for histology. CNO injections increased cFos expression in AAV-hM3Dq–injected mice compared with mCherry-injected controls (B). 3V. Data are mean ± SEM. *P < 0.05; Mann–Whitney *U* test. The underlying data for this figure are available from the Open Science Framework (https://osf.io/nmrpq/). CNO, clozapine-n-oxide; i.p., intraperitoneal; LH, lateral hypothalamic area; Nts, neurotensin; PVH, paraventricular hypothalamus; 3V, third ventricle.
(TIFF)

S3 Fig Chemoinhibition of Nts^LH^ neurons during the light period does not alter sleep-wake states or Tb. Hourly percentages of different sleep-wake states (**A**), mean Tb (**C**), and total LMA counts (**D**) for 12 h after saline or CNO (1.5 mg/kg) administration at 9:50 AM in Nts-Cre mice injected with AAV-hM4Di into the LH. Latency to NREM and REM sleep (**B**) after saline and CNO injections. Two-way RM ANOVA for “time” and “compound injected,” followed by Sidak post hoc test (*n* = 6 mice; for wake: interaction *F*(11,60) = 0.56, *P* = 0.85, compound injected *F*(11, 60) = 21.68, *P* < 0.0001, time *F*(1, 60) = 0.00018, *P* = 0.99; for NREM: interaction *F*(11,60) = 0.84, *P* = 0.60, compound injected *F*(11, 60) = 15.99, *P* < 0.0001, time *F*(1, 60) = 0.13, *P* = 0.71; for REM: interaction *F*(11,60) = 0.94, *P* = 0.51, compound injected *F*(11, 60) = 7.33, *P* < 0.0001, time *F*(1, 60) = 1.74, *P* = 0.19; for Tb: interaction *F*(11,72) = 1.36, *P* = 0.21, compound injected *F*(11, 72) = 16.14, time *F*(1, 72) = 0.51, *P* = 0.48; for LMA: interaction *F*(11,72) = 0.48, *P* = 0.91, compound injected *F*(11, 72) = 8.17, *P* < 0.0001, time *F*(1, 72) = 0.67, *P* = 0.42,). Data are mean ± SEM. *P < 0.05, **P < 0.01. The underlying data for this figure are available from the Open Science Framework (https://osf.io/nmrpq/). CNO, clozapine-n-oxide; LH, lateral hypothalamic area; LMA, locomotor activity; NREM, non-rapid eye movement; Nts, neurotensin; REM, rapid eye movement; RM, repeated measures; Tb, body temperature.
(TIFF)

S4 Fig Sleep-wake, LMA, and Tb responses to fasting. Hourly percentages of sleep-wake stages (**A**), mean Tb (**B**), and total LMA counts (**C**) for 24 h after saline injections in the fed and fasting conditions. When fasted during their active (feeding) period, mice exhibit increased wake, hyperactivity, and hyperthermia initially (food foraging), which was followed by a period of increased sleep and hypothermia (about 15 h after). Two-way RM ANOVA for the first 12 h after treatment for “time” and “compound injected,” followed by Sidak post hoc test (*n* = 7 mice; for wake: interaction *F*(23,144) = 3.14, *P* < 0.0001, compound injected *F*(23, 144) = 10.84, *P* < 0.0001, time *F*(1, 144) = 0.69, *P* = 0.41; for NREM: interaction *F*(23,144) = 3.45, *P* < 0.0001, compound injected *F*(23, 144) = 9.78, *P* < 0.0001, time *F*(1, 144) = 0.0082, *P* = 0.93; for REM: interaction *F*(23,144) = 0.82, *P* = 0.70, compound injected *F*(23, 144) = 7.51, *P* < 0.0001, time *F*(1, 144) = 20.25, *P* < 0.0001; for Tb: interaction *F*(23,144) = 3.77, *P* < 0.0001, compound injected *F*(23, 144) = 10.74, *P* < 0.0001, time *F*(1, 144) = 164.9, *P* < 0.0001; for LMA: interaction *F*(23,144) = 2.25, *P* = 0.0021, compound injected *F*(23, 144) = 4.21, *P* < 0.0001, time *F*(1, 144) = 0.082, *P* = 0.78). Data are mean ± SEM. *P < 0.05, **P < 0.01. The underlying data for this figure are available from the Open Science Framework (https://osf.io/nmrpq/). LMA, locomotor activity; NREM, non-rapid eye movement; REM, rapid eye movement; RM, repeated measures; Tb, body temperature.
(TIFF)

S1 Table Bout numbers and durations of individual sleep-wake states after saline and CNO injections during the light period into the Nts-Cre mice expressing hM3Dq in Nts^LH^ neurons. Data are mean ± SEM. *P<0.05; **P<0.01. CNO, clozapine-n-oxide; LH, lateral hypothalamic area; Nts, neurotensin.
(DOCX)

S2 Table Bout numbers and durations of individual sleep-wake states after saline and CNO injections during the dark period into the Nts-Cre mice expressing hM3Dq in Nts^LH^ neurons. Data are mean ± SEM. *P < 0.05; **P < 0.01. CNO, clozapine-n-oxide; LH, lateral hypothalamic area; Nts, neurotensin.
(DOCX)

S3 Table Bout numbers and durations of individual sleep-wake states after saline and CNO injections during the light period into the Nts-Cre mice expressing hM4Di in Nts^LH^ neurons. Data are mean ± SEM. *P < 0.05; **P < 0.01. CNO, clozapine-n-oxide; LH, lateral hypothalamic area; Nts, neurotensin.
(DOCX)

S4 Table Bout numbers and durations of individual sleep-wake states after saline and CNO injections during the dark period into the Nts-Cre mice expressing hM4Di in Nts^LH^ neurons. Data are mean ± SEM. *P < 0.05, **P < 0.01. CNO, clozapine-n-oxide; LH, lateral hypothalamic area.
(DOCX)

S5 Table Bout numbers and durations of individual sleep-wake states after saline and CNO injections and cage exchange in the Nts-Cre mice expressing hM4Di in Nts^LH^ neurons. Data are mean ± SEM. *P < 0.05, **P < 0.01. CNO, clozapine-n-oxide; LH, lateral hypothalamic area; Nts, neurotensin.
(DOCX)

S6 Table Bout numbers and durations of individual sleep-wake states after saline/CNO injections + fasting in the Nts-Cre mice expressing hM4Di in Nts^LH^ neurons. Data are mean ± SEM. CNO, clozapine-n-oxide; LH, lateral hypothalamic area; Nts, neurotensin.
(DOCX)

## Abbreviations

AAV: adeno-associated virus
AAV-ChR2: AAV8-EF1α-DIO-ChR2-mCherry
AAV-hM3Dq: AAV8-hSyn-DIO-hM3Dq-mCherry
AAV-mCherry: AAV8-DIO-hsyn-mCherry
ACSF: artificial cerebrospinal fluid
BNST: bed nucleus of stria terminalis
ChR2: channelrhodospsin-2
CNO: clozapine-n-oxide
DAB: diaminobenzidine
DMH: dorsomedial hypothalamus
DREADD: designer receptor exclusively activated by designer drugs
EEG: electroencephalography
EMG: electromyography
GFP: green fluorescent protein
i.p: intraperitoneal
LC: locus coeruleus
LH: lateral hypothalamic area
LMA: locomotor activity
MCH: melanin-concentrating hormone
NREM: non-rapid eye movement
Nts: neurotensin
Nts-GFP: Nts-Cre::L10-GFP
PB: parabrachial nucleus
Ppy: parapyramidal region
PVH: paraventricular nucleus
REM: rapid eye movement
RPa: raphe Pallidus
Tb: body temperature
vlPAG: ventrolateral periaqueductal gray
VLPO: ventrolateral preoptic area
VTA: ventral tegmental area
WPRE: woodchuck hepatitis virus posttranscriptional control element
WT: wild-type
