# Supplementary figures and images for: Lateral hypothalamic neurotensin neurons promote arousal and hyperthermia
Source: PLoS Biol. 2019 Mar 20;17(3):e3000172. doi: 10.1371/journal.pbio.3000172 (PMC6426208; doi:10.1371/journal.pbio.3000172)

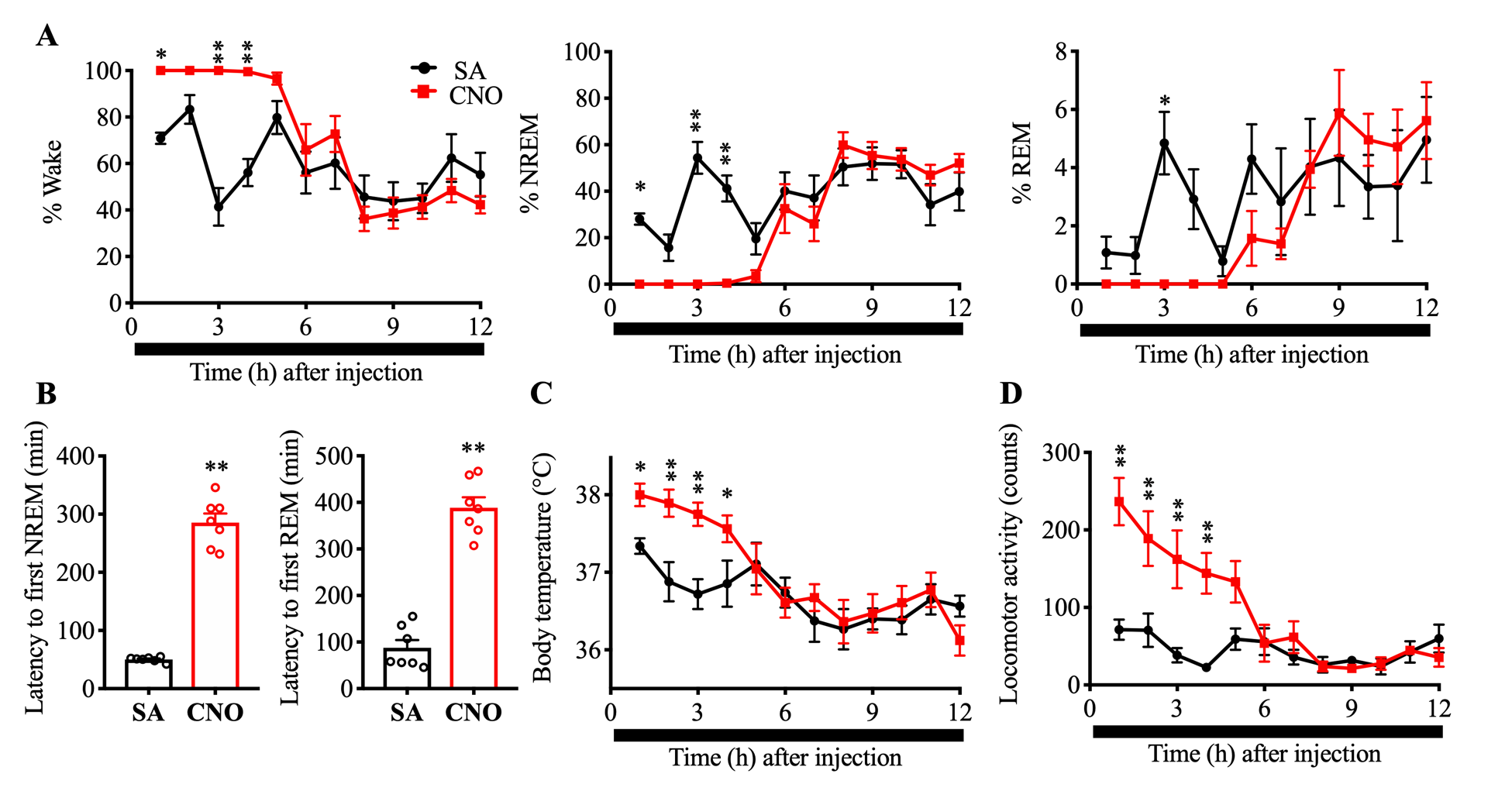

Supplement: S1 Fig — Hourly percentages of different sleep-wake states (A), mean Tb (C), and total LMA counts (D) for the 12 h after saline or CNO (0.3 mg/kg) administration at 06:50 PM in Nts-Cre mice injected with AAV-hM3Dq into the LH. Latency to NREM and REM sleep (B) after saline and CNO injections. Two-way RM ANOVA for “time” and “compound injected,” followed by Sidak post hoc test (n = 7 mice; for wake: interaction F(11,72) = 5.53, P < 0.0001, compound injected F(11, 72) = 15.45, P < 0.0001, time F(1, 72) = 17.54, P < 0.0001; for NREM: interaction F(11,72) = 5.84, P < 0.0001, compound injected F(11, 72) = 15.68, P < 0.0001, time F(1, 72) = 19.05, P < 0.0001; for REM: interaction F(11,72) = 2.00, P = 0.041, compound injected F(11, 72) = 4.64, P < 0.0001, time F(1, 72) = 3.96, P < 0.051; for Tb: interaction F(11,72) = 4.28, P < 0.0001, compound injected F(11, 72) = 6.05, P < 0.0001, time F(1, 72) = 21.64, P < 0.0001; for LMA: interaction F(11,72) = 6.76, P < 0.0001, compound injected F(11, 72) = 9.97, P < 0.0001, time F(1, 72) = 45.09, P < 0.0001). Data are mean ± SEM. *P < 0.05, **P < 0.01. The underlying data for this figure are available from the Open Science Framework (https://osf.io/nmrpq/). CNO, clozapine-n-oxide; LH, lateral hypothalamic area; LMA, locomotor activity; NREM, non-rapid eye movement; Nts, neurotensin; REM, rapid eye movement; RM, repeated measures; Tb, body temperature. (TIFF) [file pbio.3000172.s001.tiff]

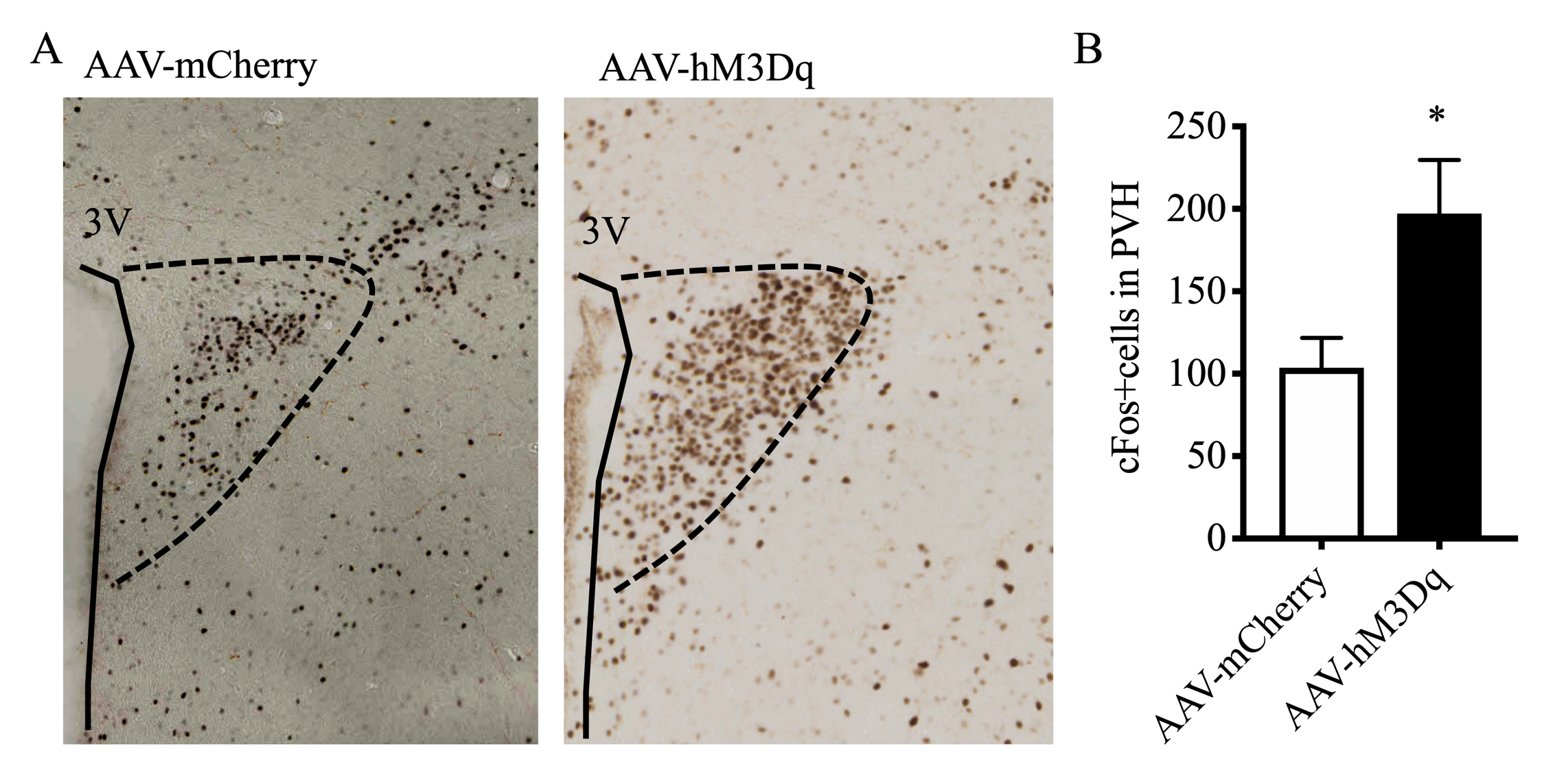

Supplement: S2 Fig — Representative brain sections at the level of PVH (marked by dashed lines) labeled for cFos (black dots) from Nts-Cre mice injected with AAV-hM3Dq or AAV-mCherry into the LH (A). These mice were injected with CNO i.p. 2.5 h before they were killed for histology. CNO injections increased cFos expression in AAV-hM3Dq–injected mice compared with mCherry-injected controls (B). 3V. Data are mean ± SEM. *P < 0.05; Mann–Whitney U test. The underlying data for this figure are available from the Open Science Framework (https://osf.io/nmrpq/). CNO, clozapine-n-oxide; i.p., intraperitoneal; LH, lateral hypothalamic area; Nts, neurotensin; PVH, paraventricular hypothalamus; 3V, third ventricle. (TIFF) [file pbio.3000172.s002.tiff]

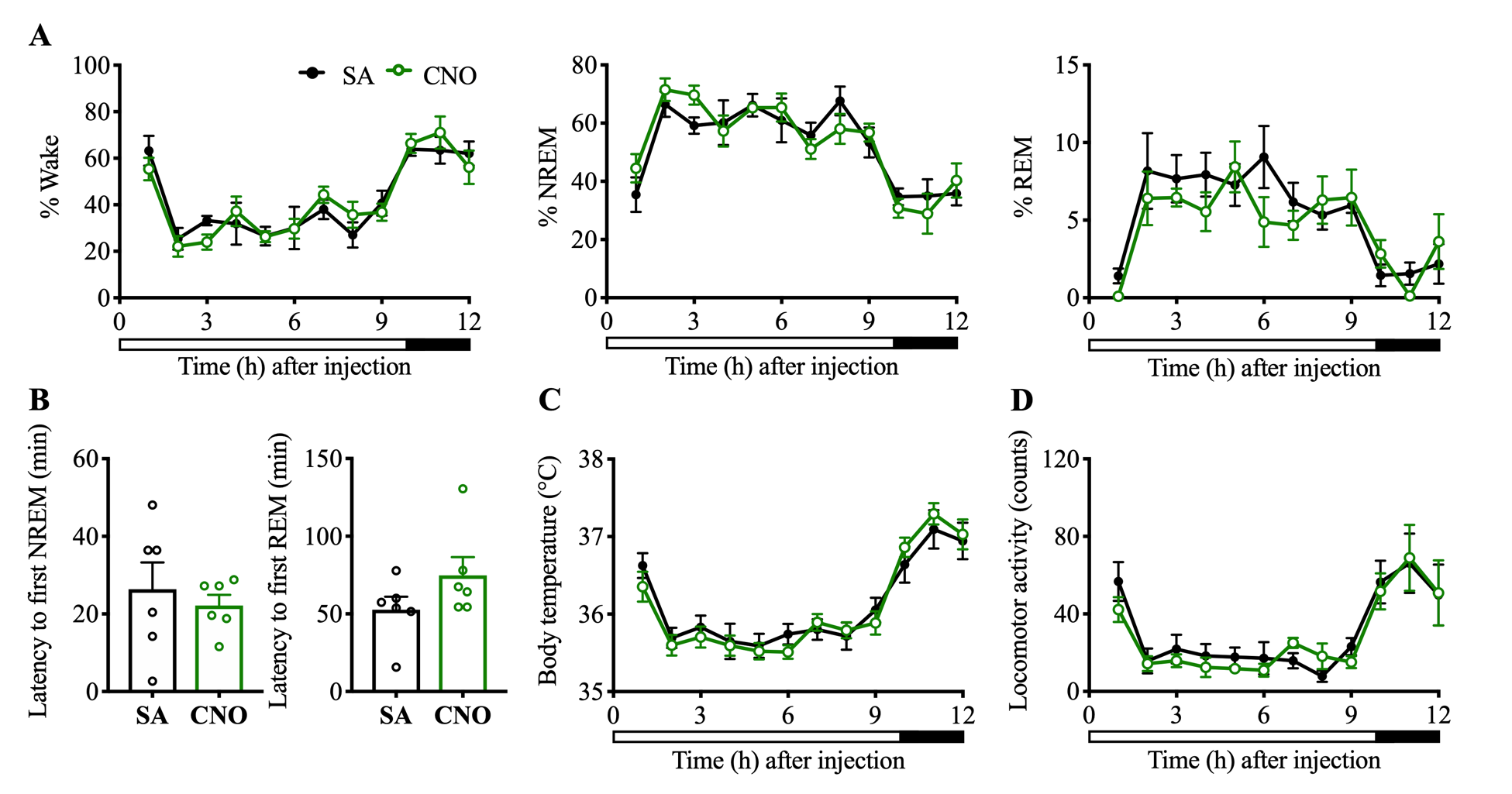

Supplement: S3 Fig — Hourly percentages of different sleep-wake states (A), mean Tb (C), and total LMA counts (D) for 12 h after saline or CNO (1.5 mg/kg) administration at 9:50 AM in Nts-Cre mice injected with AAV-hM4Di into the LH. Latency to NREM and REM sleep (B) after saline and CNO injections. Two-way RM ANOVA for “time” and “compound injected,” followed by Sidak post hoc test (n = 6 mice; for wake: interaction F(11,60) = 0.56, P = 0.85, compound injected F(11, 60) = 21.68, P < 0.0001, time F(1, 60) = 0.00018, P = 0.99; for NREM: interaction F(11,60) = 0.84, P = 0.60, compound injected F(11, 60) = 15.99, P < 0.0001, time F(1, 60) = 0.13, P = 0.71; for REM: interaction F(11,60) = 0.94, P = 0.51, compound injected F(11, 60) = 7.33, P < 0.0001, time F(1, 60) = 1.74, P = 0.19; for Tb: interaction F(11,72) = 1.36, P = 0.21, compound injected F(11, 72) = 16.14, time F(1, 72) = 0.51, P = 0.48; for LMA: interaction F(11,72) = 0.48, P = 0.91, compound injected F(11, 72) = 8.17, P < 0.0001, time F(1, 72) = 0.67, P = 0.42,). Data are mean ± SEM. *P < 0.05, **P < 0.01. The underlying data for this figure are available from the Open Science Framework (https://osf.io/nmrpq/). CNO, clozapine-n-oxide; LH, lateral hypothalamic area; LMA, locomotor activity; NREM, non-rapid eye movement; Nts, neurotensin; REM, rapid eye movement; RM, repeated measures; Tb, body temperature. (TIFF) [file pbio.3000172.s003.tiff]

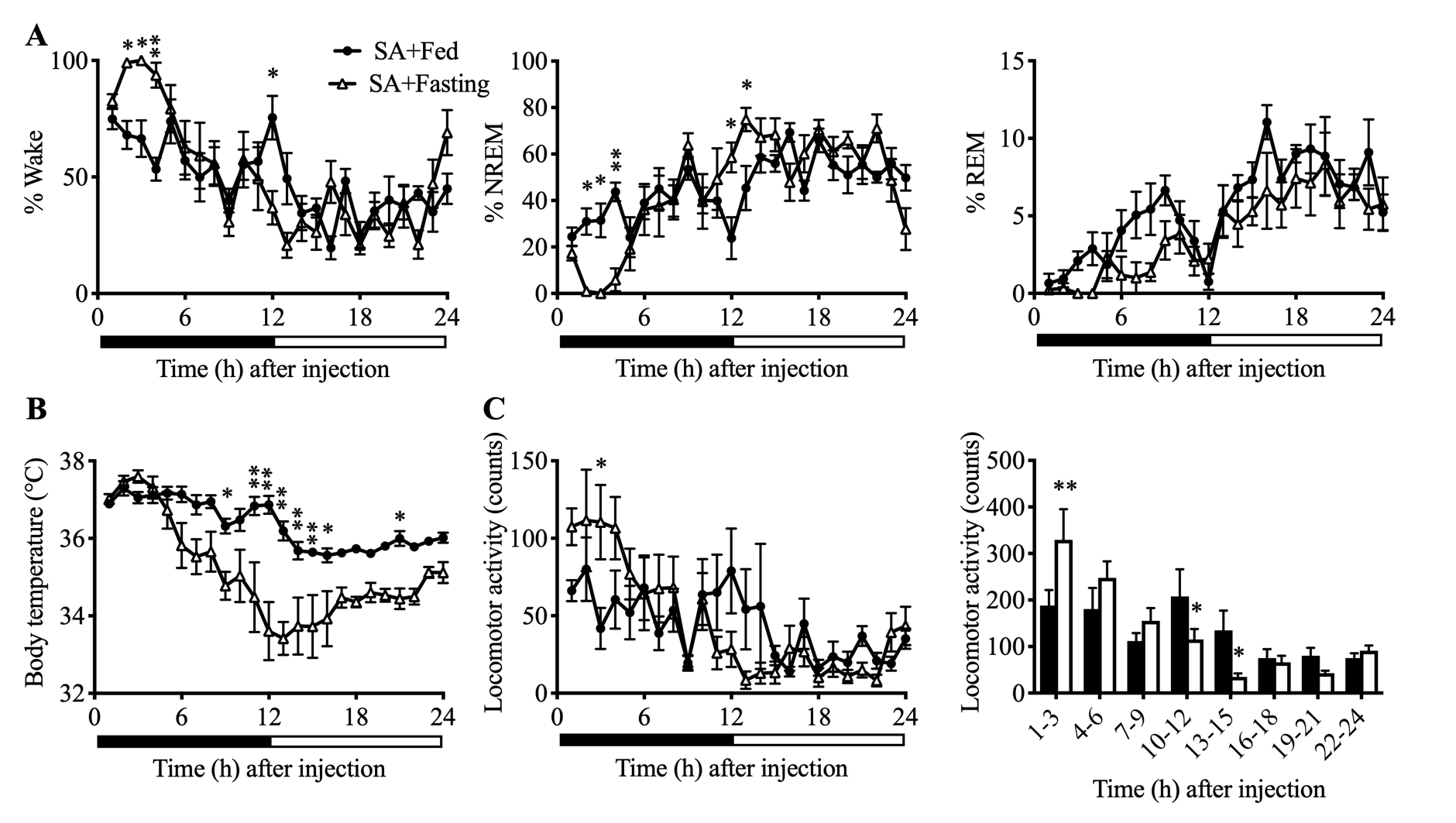

Supplement: S4 Fig — Hourly percentages of sleep-wake stages (A), mean Tb (B), and total LMA counts (C) for 24 h after saline injections in the fed and fasting conditions. When fasted during their active (feeding) period, mice exhibit increased wake, hyperactivity, and hyperthermia initially (food foraging), which was followed by a period of increased sleep and hypothermia (about 15 h after). Two-way RM ANOVA for the first 12 h after treatment for “time” and “compound injected,” followed by Sidak post hoc test (n = 7 mice; for wake: interaction F(23,144) = 3.14, P < 0.0001, compound injected F(23, 144) = 10.84, P < 0.0001, time F(1, 144) = 0.69, P = 0.41; for NREM: interaction F(23,144) = 3.45, P < 0.0001, compound injected F(23, 144) = 9.78, P < 0.0001, time F(1, 144) = 0.0082, P = 0.93; for REM: interaction F(23,144) = 0.82, P = 0.70, compound injected F(23, 144) = 7.51, P < 0.0001, time F(1, 144) = 20.25, P < 0.0001; for Tb: interaction F(23,144) = 3.77, P < 0.0001, compound injected F(23, 144) = 10.74, P < 0.0001, time F(1, 144) = 164.9, P < 0.0001; for LMA: interaction F(23,144) = 2.25, P = 0.0021, compound injected F(23, 144) = 4.21, P < 0.0001, time F(1, 144) = 0.082, P = 0.78). Data are mean ± SEM. *P < 0.05, **P < 0.01. The underlying data for this figure are available from the Open Science Framework (https://osf.io/nmrpq/). LMA, locomotor activity; NREM, non-rapid eye movement; REM, rapid eye movement; RM, repeated measures; Tb, body temperature. (TIFF) [file pbio.3000172.s004.tiff]
