# Supplementary material for: Lateral hypothalamic neurotensin neurons promote arousal and hyperthermia
Source: PLoS Biol. 2019 Mar 20;17(3):e3000172. doi: 10.1371/journal.pbio.3000172 (PMC6426208; doi:10.1371/journal.pbio.3000172)
Supplement: S1 Table — Data are mean ± SEM. *P<0.05; **P<0.01. CNO, clozapine-n-oxide; LH, lateral hypothalamic area; Nts, neurotensin. (DOCX) [file pbio.3000172.s005.docx]

|  | | Number of bouts | | Mean bout duration(s) | |
| --- | --- | --- | --- | --- | --- |
|  |  | Post-saline | Post-CNO | Post-saline | Post-CNO |
| Wake | 1-3 h | 30.63 ± 1.79 | 1.00 ± 0.00** | 162.50 ± 13.85 | 10800.00 ± 0.00** |
|  | 4-6 h | 38.00 ± 2.87 | 22.00 ± 6.23* | 91.63 ± 11.55 | 2103.17 ± 1742.15 |
|  | 7-9 h | 38.00 ± 3.16 | 31.00 ± 4.87 | 110.75 ± 14.99 | 147.50 ± 49.86 |
|  | 10-12 h | 27.38 ± 2.25 | 39.83 ± 4.79* | 250.88 ± 25.21 | 120.67 ± 20.20** |
| NREM | 1-3 h | 30.50 ± 1.88 | 0.00 ± 0.00** | 183.63 ± 17.18 | 0.00 ± 0.00** |
|  | 4-6 h | 38.63 ± 2.83 | 21.67 ± 6.30* | 180.75 ± 17.39 | 144.20 ± 11.52 |
|  | 7-9 h | 38.38 ± 3.11 | 31.33 ± 5.10 | 168.00 ± 18.25 | 219.67 ± 27.70 |
|  | 10-12 h | 27.63 ± 2.10 | 39.67 ± 4.96** | 149.25 ± 14.87 | 154.17 ± 24.02 |
| REM | 1-3 h | 8.63 ± 1.24 | 0.00 ± 0.00** | 57.75 ± 5.94 | 0.00 ± 0.00** |
|  | 4-6 h | 13.13 ± 2.00 | 4.83 ± 2.29* | 52.88 ± 3.18 | 72.50 ± 17.29* |
|  | 7-9 h | 11.50 ± 2.13 | 11.33 ± 2.76 | 55.38 ± 4.13 | 76.67 ± 13.26 |
|  | 10-12 h | 5.25 ± 0.84 | 10.67 ± 2.22** | 50.13 ± 4.85 | 67.67 ± 4.40* |
