# Supplementary material for: Lateral hypothalamic neurotensin neurons promote arousal and hyperthermia
Source: PLoS Biol. 2019 Mar 20;17(3):e3000172. doi: 10.1371/journal.pbio.3000172 (PMC6426208; doi:10.1371/journal.pbio.3000172)
Supplement: S2 Table — Data are mean ± SEM. *P < 0.05; **P < 0.01. CNO, clozapine-n-oxide; LH, lateral hypothalamic area; Nts, neurotensin. (DOCX) [file pbio.3000172.s006.docx]

|  | | Number of bouts | | Mean bout duration(s) | |
| --- | --- | --- | --- | --- | --- |
|  |  | Post-saline | Post-CNO | Post-saline | Post-CNO |
| Wake | 1-3 h | 27.67 ± 2.17 | 1.00 ± 0.00** | 269.00 ± 40.93 | 10800.00 ± 0.00** |
|  | 4-6 h | 28.83 ± 2.52 | 11.00 ± 2.12** | 238.83 ± 24.98 | 1555.86 ± 695.12 |
|  | 7-9 h | 33.17 ± 2.70 | 28.57 ± 2.59 | 163.17 ± 27.67 | 195.00 ± 32.49 |
|  | 10-12 h | 29.83 ± 4.48 | 34.00 ± 2.50 | 268.17 ± 105.46 | 153.71 ± 18.51 |
| NREM | 1-3 h | 27.33 ± 2.43 | 0.00 ± 0.00** | 130.17 ± 5.60 | 0.00 ± 0.00** |
|  | 4-6 h | 28.67 ± 2.65 | 10.43 ± 2.22** | 140.83 ± 9.74 | 155.00 ± 22.69 |
|  | 7-9 h | 33.00 ± 2.77 | 28.29 ± 2.78 | 164.67 ± 13.81 | 192.00 ± 16.53 |
|  | 10-12 h | 29.83 ± 4.53 | 33.71 ± 2.37 | 158.67 ± 12.16 | 159.29 ± 10.94 |
| REM | 1-3 h | 4.33 ± 1.52 | 0.00 ± 0.00* | 49.60 ± 7.60 | 0.00 ± 0.00* |
|  | 4-6 h | 5.33 ± 1.28 | 1.43 ± 0.72* | 47.60 ± 6.94 | 74.00 ± 15.88 |
|  | 7-9 h | 6.50 ± 1.96 | 7.71 ± 1.39 | 54.00 ± 7.35 | 63.29 ± 8.26 |
|  | 10-12 h | 6.17 ± 1.80 | 9.43 ± 1.76 | 66.67 ± 12.58 | 52.29 ± 6.14 |
