# Supplementary material for: Lateral hypothalamic neurotensin neurons promote arousal and hyperthermia
Source: PLoS Biol. 2019 Mar 20;17(3):e3000172. doi: 10.1371/journal.pbio.3000172 (PMC6426208; doi:10.1371/journal.pbio.3000172)
Supplement: S3 Table — Data are mean ± SEM. *P < 0.05; **P < 0.01. CNO, clozapine-n-oxide; LH, lateral hypothalamic area; Nts, neurotensin. (DOCX) [file pbio.3000172.s007.docx]

|  | | Number of bouts | | Mean bout duration(s) | |
| --- | --- | --- | --- | --- | --- |
|  |  | Post-saline | Post-CNO | Post-saline | Post-CNO |
| Wake | 1-3 h | 42.17 ± 6.07 | 32.17 ± 2.75 | 113.33 ± 15.20 | 117.17 ± 12.74 |
|  | 4-6 h | 44.67 ± 3.64 | 39.33 ± 5.74 | 71.33 ± 11.02 | 102.83 ± 25.20 |
|  | 7-9 h | 42.83 ± 1.97 | 47.00 ± 5.13 | 88.17 ± 11.45 | 93.17 ± 8.58 |
|  | 10-12 h | 30.83 ± 3.59 | 37.00 ± 4.91 | 235.83 ± 26.87 | 230.67 ± 67.55 |
| NREM | 1-3 h | 42.17 ± 5.88 | 32.33 ± 2.75 | 151.33 ± 20.99 | 212.83 ± 19.10 |
|  | 4-6 h | 45.33 ± 3.54 | 40.17 ± 5.69 | 152.83 ± 18.73 | 184.17 ± 24.40 |
|  | 7-9 h | 42.33 ± 2.16 | 47.17 ± 5.08 | 153.00 ± 16.64 | 133.67 ± 14.97 |
|  | 10-12 h | 30.83 ± 3.60 | 36.67 ± 4.70 | 132.33 ± 19.49 | 103.17 ± 9.68 |
| REM | 1-3 h | 11.00 ± 1.69 | 8.00 ± 0.52 | 57.83 ± 3.26 | 59.50 ± 9.82 |
|  | 4-6 h | 15.17 ± 1.90 | 10.00 ± 1.06 | 60.5 ± 2.92 | 68.00 ± 3.55 |
|  | 7-9 h | 12.83 ± 1.35 | 11.00 ± 1.44 | 51.17 ± 5.94 | 54.50 ± 5.16 |
|  | 10-12 h | 2.67 ± 1.20 | 3.83 ± 1.01 | 66.80 ± 14.51 | 64.50 ± 9.89 |
