# Supplementary material for: Lateral hypothalamic neurotensin neurons promote arousal and hyperthermia
Source: PLoS Biol. 2019 Mar 20;17(3):e3000172. doi: 10.1371/journal.pbio.3000172 (PMC6426208; doi:10.1371/journal.pbio.3000172)
Supplement: S4 Table — Data are mean ± SEM. *P < 0.05, **P < 0.01. CNO, clozapine-n-oxide; LH, lateral hypothalamic area. (DOCX) [file pbio.3000172.s008.docx]

|  | | Number of bouts | | Mean bout duration(s) | |
| --- | --- | --- | --- | --- | --- |
|  |  | Post-saline | Post-CNO | Post-saline | Post-CNO |
| Wake | 1-3 h | 29.67 ± 5.17 | 29.71 ± 3.78 | 314.67 ± 81.65 | 245.29 ± 41.68 |
|  | 4-6 h | 37.50 ± 5.94 | 28.29 ± 3.26 | 229.00 ± 78.36 | 251.00 ± 37.04 |
|  | 7-9 h | 35.67 ± 3.21 | 34.14 ± 3.26 | 159.33 ± 23.00 | 175.43 ± 23.09 |
|  | 10-12 h | 31.00 ± 5.74 | 33.86 ± 2.43 | 357.83 ± 155.79 | 165.29 ± 25.69 |
| NREM | 1-3 h | 29.00 ± 5.29 | 29.14 ± 3.86 | 117.50 ± 12.29 | 152.86 ± 18.08 |
|  | 4-6 h | 37.17 ± 5.82 | 28.14 ± 3.47 | 107.17 ± 6.21 | 158.00 ± 18.72 |
|  | 7-9 h | 36.00 ± 3.28 | 33.85 ± 3.29 | 139.17 ± 13.91 | 143.71 ± 20.50 |
|  | 10-12 h | 30.67 ± 5.83 | 33.86 ± 2.60 | 105.67 ± 7.95 | 149.71 ± 14.79 |
| REM | 1-3 h | 3.33 ± 1.43 | 2.85 ± 0.55 | 47.60 ± 2.67 | 61.43 ± 10.79 |
|  | 4-6 h | 6.17 ± 1.62 | 4.57 ± 1.04 | 56.40 ± 6.35 | 63.43 ± 3.60 |
|  | 7-9 h | 8.33 ± 0.71 | 7.14 ± 0.91 | 76.00 ± 12.90 | 75.29 ± 2.07 |
|  | 10-12 h | 4.83 ± 1.47 | 8.14 ± 1.44 | 59.17 ± 10.31 | 68.43 ± 6.91 |
