# Supplementary material for: Lateral hypothalamic neurotensin neurons promote arousal and hyperthermia
Source: PLoS Biol. 2019 Mar 20;17(3):e3000172. doi: 10.1371/journal.pbio.3000172 (PMC6426208; doi:10.1371/journal.pbio.3000172)
Supplement: S5 Table — Data are mean ± SEM. *P < 0.05, **P < 0.01. CNO, clozapine-n-oxide; LH, lateral hypothalamic area; Nts, neurotensin. (DOCX) [file pbio.3000172.s009.docx]

|  | | Number of bouts | | Mean bout duration(s) | |
| --- | --- | --- | --- | --- | --- |
|  |  | Post-saline | Post-CNO | Post-saline | Post-CNO |
| Wake | 1-3 h | 1.00 ± 0.00 | 11.00 ± 2.59* | 10800.00 ± 0.00 | 2445.33 ± 1674.25** |
|  | 4-6 h | 22.43 ± 3.82 | 32.67 ± 2.64* | 479.86 ± 227.78 | 130.00 ± 15.91 |
|  | 7-9 h | 30.29 ± 3.04 | 36.33 ± 4.38 | 153.00 ± 19.85 | 132.83 ± 28.52 |
|  | 10-12 h | 16.86 ± 3.28 | 26.17 ± 4.37 | 1136.29 ± 699.82 | 940.17 ± 521.30 |
| NREM | 1-3 h | 0.00 ± 0.00 | 10.33 ± 2.76* | 0.00 ± 0.00 | 152.20 ± 37.16* |
|  | 4-6 h | 22.29 ± 3.88 | 33.00 ± 2.46* | 213.57 ± 35.92 | 187.00 ± 16.57 |
|  | 7-9 h | 30.29 ± 3.11 | 36.50 ± 4.33 | 194.43 ± 18.65 | 167.67 ± 20.46 |
|  | 10-12 h | 16.14 ± 3.23 | 18.50 ± 4.55 | 157.43 ± 12.98 | 121.50 ± 4.80* |
| REM | 1-3 h | 0.00 ± 0.00 | 1.33 ± 0.61 | 0.00 ± 0.00 | 74.25 ± 12.94* |
|  | 4-6 h | 7.00 ± 1.86 | 9.17 ± 0.79 | 67.00 ± 8.78 | 78.67 ± 9.29 |
|  | 7-9 h | 11.86 ± 1.32 | 12.67 ± 2.18 | 62.29 ± 2.93 | 71.00 ± 7.37 |
|  | 10-12 h | 3.86 ± 0.80 | 4.00 ± 1.10 | 72.50 ± 8.29 | 64.60 ± 5.00 |
