# Supplementary material for: Lateral hypothalamic neurotensin neurons promote arousal and hyperthermia
Source: PLoS Biol. 2019 Mar 20;17(3):e3000172. doi: 10.1371/journal.pbio.3000172 (PMC6426208; doi:10.1371/journal.pbio.3000172)
Supplement: S6 Table — Data are mean ± SEM. CNO, clozapine-n-oxide; LH, lateral hypothalamic area; Nts, neurotensin. (DOCX) [file pbio.3000172.s010.docx]

|  | | Number of bouts | | Mean bout duration(s) | |
| --- | --- | --- | --- | --- | --- |
|  |  | Post-saline | Post-CNO | Post-saline | Post-CNO |
| Wake | 1-3 h | 5.00 ± 1.04 | 5.43 ± 1.04 | 2245.86 ± 322.93 | 3077.57 ± 1304.52 |
|  | 4-6 h | 15.14 ± 4.83 | 8.57 ± 5.90 | 2215.43 ± 1449.30 | 7028.14 ± 1876.26 |
|  | 7-9 h | 30.57 ± 4.15 | 20.57 ± 3.61 | 218.00 ± 59.14 | 581.43 ± 252.80 |
|  | 10-12 h | 31.00 ± 5.52 | 44.43 ± 6.11 | 223.57 ± 58.76 | 122.29 ± 27.14 |
|  | 13-15 h | 55.29 ± 6.09 | 54.14 ± 3.33 | 54.57 ± 8.50 | 68.00 ± 8.60 |
|  | 16-18 h | 49.86 ± 4.74 | 56.00 ± 5.92 | 74.29 ± 7.16 | 72.29 ± 12.65 |
|  | 19-21 h | 51.29 ± 5.24 | 50.67 ± 5.18 | 73.86 ± 10.11 | 72.17 ± 8.59 |
|  | 22-24 h | 44.71 ± 6.68 | 47.86 ± 5.17 | 130.71 ± 30.12 | 103.71 ± 20.59 |
| NREM | 1-3 h | 4.14 ± 0.64 | 4.57 ± 1.13 | 168.86 ± 26.05 | 183.83 ± 52.25 |
|  | 4-6 h | 14.57 ± 4.85 | 7.86 ± 6.05 | 161.33 ± 13.06 | 283.33 ± 74.47 |
|  | 7-9 h | 30.57 ± 4.40 | 20.57 ± 3.57 | 175.00 ± 14.02 | 149.14 ± 13.58 |
|  | 10-12 h | 31.29 ± 5.58 | 44.57 ± 6.15 | 178.57 ± 14.34 | 140.29 ± 13.91 |
|  | 13-15 h | 55.71 ± 6.36 | 54.71 ± 3.38 | 141.86 ± 14.10 | 123.00 ± 9.73 |
|  | 16-18 h | 50.14 ± 4.86 | 56.29 ± 5.98 | 138.71 ± 24.66 | 117.14 ± 12.84 |
|  | 19-21 h | 51.57 ± 5.09 | 51.00 ± 4.96 | 132.14 ± 12.34 | 132.00 ± 15.47 |
|  | 22-24 h | 44.71 ± 6.74 | 47.57 ± 5.04 | 129.00 ± 16.65 | 124.57 ± 10.31 |
| REM | 1-3 h | 0.00 ± 0.00 | 0.29 ± 0.18 | 0.00 ± 0.00 | 72.00 ± 6.41 |
|  | 4-6 h | 1.86 ± 0.63 | 1.14 ± 0.77 | 79.80 ± 18.80 | 104.50 ± 13.10 |
|  | 7-9 h | 6.14 ± 1.34 | 3.00 ± 0.69 | 89.43 ± 11.81 | 71.67 ± 10.67 |
|  | 10-12 h | 6.43 ± 1.17 | 4.14 ± 1.03 | 69.71 ± 14.60 | 83.71 ± 15.18 |
|  | 13-15 h | 10.29 ± 3.35 | 8.43 ± 1.54 | 82.14 ± 22.49 | 73.43 ± 13.19 |
|  | 16-18 h | 11.14 ± 2.38 | 10.00 ± 2.37 | 90.71 ± 17.26 | 73.43 ± 8.32 |
|  | 19-21 h | 13.43 ± 2.82 | 15.17 ± 1.52 | 68.71 ± 8.57 | 61.17 ± 6.89 |
|  | 22-24 h | 9.71 ± 2.01 | 10.57 ± 1.36 | 71.71 ± 7.42 | 68.14 ± 8.88 |
